# Stroke Survivors on Twitter: Sentiment and Topic Analysis From a Gender Perspective

**DOI:** 10.2196/14077

**Published:** 2019-08-26

**Authors:** Alejandro Garcia-Rudolph, Sara Laxe, Joan Saurí, Montserrat Bernabeu Guitart

**Affiliations:** 1 Institut Guttmann Hospital de Neurorehabilitacio Badalona Spain; 2 Universitat Autònoma de Barcelona Bellaterra (Cerdanyola del Vallès) Spain; 3 Fundació Institut d’Investigació en Ciències de la Salut Germans Trias i Pujol Badalona Spain

**Keywords:** stroke, emotions, Twitter, infodemiology, infoveillance, sentiment analysis, topic models, gender

## Abstract

**Background:**

Stroke is the worldwide leading cause of long-term disabilities. Women experience more activity limitations, worse health-related quality of life, and more poststroke depression than men. Twitter is increasingly used by individuals to broadcast their day-to-day happenings, providing unobtrusive access to samples of spontaneously expressed opinions on all types of topics and emotions.

**Objective:**

This study aimed to consider the raw frequencies of words in the collection of tweets posted by a sample of stroke survivors and to compare the posts by gender of the survivor for 8 basic emotions (anger, fear, anticipation, surprise, joy, sadness, trust and disgust); determine the proportion of each emotion in the collection of tweets and statistically compare each of them by gender of the survivor; extract the main topics (represented as sets of words) that occur in the collection of tweets, relative to each gender; and assign happiness scores to tweets and topics (using a well-established tool) and compare them by gender of the survivor.

**Methods:**

We performed sentiment analysis based on a state-of-the-art lexicon (National Research Council) with *syuzhet* R package. The emotion scores for men and women were first subjected to an F-test and then to a Wilcoxon rank sum test. We extended the emotional analysis, assigning happiness scores with the hedonometer (a tool specifically designed considering Twitter inputs). We calculated daily happiness average scores for all tweets. We created a term map for an exploratory clustering analysis using VosViewer software. We performed structural topic modelling with *stm* R package, allowing us to identify main topics by gender. We assigned happiness scores to all the words defining the main identified topics and compared them by gender.

**Results:**

We analyzed 800,424 tweets posted from August 1, 2007 to December 1, 2018, by 479 stroke survivors: Women (n=244) posted 396,898 tweets, and men (n=235) posted 403,526 tweets. The stroke survivor condition and gender as well as membership in at least 3 stroke-specific Twitter lists of active users were manually verified for all 479 participants. Their total number of tweets since 2007 was 5,257,433; therefore, we analyzed the most recent 15.2% of all their tweets. Positive emotions (anticipation, trust, and joy) were significantly higher (*P*<.001) in women, while negative emotions (disgust, fear, and sadness) were significantly higher (*P*<.001) in men in the analysis of raw frequencies and proportion of emotions. Happiness mean scores throughout the considered period show higher levels of happiness in women. We calculated the top 20 topics (with percentages and CIs) more likely addressed by gender and found that women’s topics show higher levels of happiness scores.

**Conclusions:**

We applied two different approaches—the Plutchik model and hedonometer tool—to a sample of stroke survivors’ tweets. We conclude that women express positive emotions and happiness much more than men.

## Introduction

### General Background

Tweets can contain information about the mood of their authors. Even when users are not specifically posting about their personal emotive status, the message can reflect their mood. As such, tweets are regarded as microscopic instantiations of emotions. Twitter has been extensively analyzed for health-related conditions. Nevertheless, to the best of our knowledge, no study has been conducted in chronic stroke, with a focus on the emotional aspects and topics addressed by stroke survivors.

### Stroke in Young Adults

Stroke is the third leading cause of long-term disability and one of the leading causes of depression worldwide [1]. Evidence suggests that stroke incidence in young adults is increasing in high-income countries [2]. It has been recently reported that ischemic stroke is no longer a disease affecting just elderly people, and an estimated 3.6 million young people (age<55 years) are affected each year [3]. The burden of stroke in young people may be increasing further, since multiple recent studies have reported increasing incidence of ischemic strokes, particularly at younger ages, while the incidence at older ages has been declining during the same period [4].

Globally, almost half of the entire stroke burden is on young individuals, as they have a greater likelihood to survive strokes, with long life spans ahead, and because strokes occur at younger ages in low- and middle-income countries [5]. Moreover, the overall population burden of cerebrovascular disease in young people may be underestimated, since clinically silent infarcts and white-matter changes are prevalent even in young stroke patients [6].

About one-fourth of ischemic strokes occur in working-aged individuals in high-income countries, with the incidence increasing worldwide in this age group from the 1980s to present [3].

### Gender Differences in Stroke Outcomes

After experiencing a stroke, women experience more activity limitations, worse health-related quality of life (HRQoL), and more poststroke depression than men, as recently reported in an updated systematic review of sex differences [7].

Recent research published in January 2019 in the European Journal of Neurology reported that women are twice as likely to suffer from severe depression following a stroke than men. Ayis and colleagues [8] followed the progression of symptoms over 5 years after stroke onset in 2313 people (1275 men and 1038 women) from the South London Stroke Register and found that 20% of women suffered from severe depression compared to 10% of men [8].

The higher prevalence of depression among women may reflect a higher prevalence in the general population, where depression was identified as the leading cause of disease burden in women worldwide [9].

### #Stroke

The expansion of social media has changed the way in which patients, physicians, and other health care stakeholders interact [10]. Twitter has led to the development of disease-specific communities that can categorize and aggregate their interactions using “hashtags.” These Twitter communities serve as readily accessible, no-cost platforms that provide significant educational and professional benefits.

Within stroke medicine, social media, specifically, Twitter has been recently highlighted for its potential to benefit patients, stroke organizations, and medical education [11].

The stroke-related Twitter network has been recently studied [12], through 621,653 tweets containing the #Stroke hashtag from March 20, 2012, to January 31, 2018, in relation to tweet content, activity metrics, engagement, and user characteristics. The most commonly discussed topics were prevention, diabetes, atrial fibrillation, aphasia, dementia, thrombectomy devices, thrombolysis, and tobacco. Specifically, the content of discussions included recognition of the signs of a stroke, associated risk factors (eg, atrial fibrillation, heart disease, and diabetes), and findings of peer-reviewed journals regarding stroke treatment. Tweets were mainly composed by advocacy/support organizations (21.5%), physicians (8.4%), individuals not known to be directly working in the health care industry (14.0%), other health care professionals (5.5%), organizations related to research/academia (2.3%), and academics (2.2%), while stroke patients contributed to 6.7% of tweets (n=41,822). There was a similar proportion of total tweets with the #Stroke hashtag generated by physicians (8.4%) and patients (6.7%) during the study period and apparent minimal network communication between physicians and patients, as reported in the study conclusions [12].

### Emotional Distress in the Adjustment Process for Stroke Survivors

Brennan emphasizes the importance of assumptions in adapting to the world around us. According to Brennan’s model, we each have a cognitive map or representation of the world, resulting from our social and cultural context and the accumulation of our life experience. This highly complex “assumptive world” is biologically adaptive in that it allows us to anticipate and plan for the future [13].

In the case of a typical stroke patient, their assumptive world will almost always be challenged or disconfirmed by the experience of stroke and its immediate repercussions [14]. As Brennan states, “adjusting core assumptions involves huge amounts of cognitive processing and emotional distress, this often leads to acute emotional difficulties, such as feelings of confusion, loss, sadness and anger.”

Moreover, the experience of stroke and disability may also confirm previously held negative beliefs for some individuals (eg, “I am worthless” or “Others see me as weak”) and may lead to emotional distress in this manner [14].

### Spontaneous, Emotional Language, and Everyday Topic Discussions on Twitter

Over the last few years, Twitter has become a notable data source in sociolinguistics, as it captures opinions and sentiments on a wide range of topics. Although Twitter users are a self-selected group, it has been argued that analyses of Twitter data produce results congruent with those obtained using standard research methods and data sources [15].

Considering the frequent use of emotional language in tweets that relate to everyday experiences [16], for a large proportion of the population, Twitter provides unobtrusive access to time-sensitive and ecologically valid samples of spontaneously expressed emotions [17].

Sentiment analysis in the health care setting is not a new phenomenon, for example, in previous research, greater positive sentiment within discharge summaries was associated with a significantly decreased risk of readmission [18].

### This Study

In the following subsections, we describe the specific characteristics and objectives of our study.

#### Twitter Lists

Previous studies have shown that topical experts are often the primary drivers of interesting discussions on Twitter [19]. In contrast to random sampling for gathering Twitter data, alternative sampling methods have been put forward; one of them proposed to retrieve content only from topical experts, that is, Twitter users whose followers consider them to be knowledgeable on some topic, to reduce the number of unwanted tweets in the sampled data while still gathering useful tweets related to a specific topic. The key challenge, however, lies in identifying a good set of experts [20].

Twitter users can organize the accounts that they follow into Twitter user lists. These lists are used in a variety of ways. In some cases, they may correspond to personal lists of a given user’s friends and families, but frequently, lists are employed to group together Twitter accounts based on a common topic or theme. In this way, every Twitter user can effectively become a community curator. Therefore, previous research has proposed that we consider a Twitter user a “topical expert” if the user belongs to several lists on a particular topic [20].

In our study, we propose to take advantage of user lists in the field of stroke. To the best of our knowledge, lists have not been used in studies related to chronic health conditions.

#### Plutchik’s Human Emotions

Currently, there is no single accepted psychological theory of basic human emotions; nevertheless, there is an agreement that a simple positive-negative dichotomy is not enough to capture the full range of emotions [21].

In this work, we use the Plutchik [22] approach, which postulates the following eight basic human emotions: joy, sadness, anger, fear, trust, disgust, anticipation, and surprise. There have been extensive applications of this approach, for example, the National Research Council (NRC) Word-Emotion Association Lexicon, which contains 10,170 lexical items that are coded for Plutchik’s basic human emotions [23], and has been applied in several sentiment analysis studies [24].

Plutchik’s categories also have the advantage of providing a balanced list of positive (trust, joy, anger, and anticipation) and negative (disgust, sadness, fear, and surprise) emotions, which, to the best of our knowledge, have not been applied in chronic conditions, in general, or stroke, in particular.

### Hedonometer

After performing emotional analysis based on Putchik’s model, we propose another point of view, by assigning happiness scores to tweets with the hedonometer tool. The hedonometer [25] was developed from Twitter, Google Books, music lyrics, and the New York Times for measuring expressed happiness—positive and negative sentiment—in large-scale text corpora. Since its development, the hedonometer has been applied to studies on predictive markers of depression on Instagram [26] or the climate change sentiment on Twitter [27]. The hedonometer calculates a happiness score based on the happiness of the individual words used in the text. A total of 10,222 of the most frequently used English words in four disparate corpora were given happiness ratings using Amazon’s Mechanical Turk online marketplace.

### Adding Covariate Information With Structural Topic Models

Although Latent Dirichlet Allocation (LDA) is, perhaps, the most common form of topic modeling, a number of associated techniques now exist, including dynamic topic models, correlated topic models, and hierarchical topic models. One of the most increasingly popular techniques to emerge in recent years, however, is structural topic modeling (STM). STM provides a flexible way to incorporate “metadata” associated with the text, such as when the text was written, where (eg, which country) it was written, who wrote it, and characteristics of the author, into the analysis using document-level covariates. In turn, it allows analysis of relationships between metadata and topics in the text corpus.

### Study Objectives

As Brennan states [13], the adjusting process involves huge amounts of emotional distress. This often leads to acute emotional difficulties such as feelings of confusion, loss, sadness, and anger. Considering that women experience more activity limitations, worse health-related quality of life, and more poststroke depression, in this study, we propose to take advantage of unobtrusive access to samples of spontaneously expressed emotions and opinions provided by Twitter and to analyze them from a gender perspective using two different, well-established approaches (Plutchik model and the hedonometer tool), with the following specific aims:

- To compare tweets by gender of stroke survivor for the 8 basic emotions (anger, fear, anticipation, surprise, joy, sadness, trust, and disgust) while considering the raw frequencies of words in the collection of tweets posted by a stroke survivors’ sample.
- To determine the proportion of each emotion in the collection of tweets and statistically compare each of them by gender. This measurement thus allows us to track the proportion of each emotion for each individual tweet and is less affected by single outliers.
- To extract the main topics (represented as sets of words) that occur in the collection of tweets, related to each gender.
- To assign happiness scores to tweets and topics (using the hedonometer) and compare them by gender.

## Methods

### Data Collection

We considered the network analysis from previous research [12] (see #Stroke in the Introduction) as the starting point. Node size is related to user influence, which is directly correlated to the amount a user is mentioned. The top identified nodes and their corresponding number of followers are as follows: @TheStrokeAssoc (102 million), @signagnststroke (68 million), @StrokeHope (93.8 million), @PeterCoghlan1 (7.2 million), @strokefdn (11.5 million), @StrokeAssocNW (5.5 million), @StrokeAHA_ASA (10.6 million), and @HeartandStroke (45.3 million), @HeartandStroke (45.3 million).

Twitter data collection was performed using the *rtweet* R package [28] via Twitter's REST (representational state transfer) and stream application program interfaces (APIs). We initially applied the *lists_users* function to obtain all lists that the top nodes subscribe to, including their own. Subsequently, we used the *lists_members* function to obtain Twitter list members (users on a given list). To retain a list member, we imposed the condition that it should appear in at least 3 different lists.

For each identified user, we retrieved tweets with the get_timelines() function (it retrieves the most recent 3200 tweets for each Twitter user, without any time restriction). We collected 1,300,845 tweets from a thousand users and further classified them in tweets from particulars (woman or man) and from organizations (institutions and associations); the last step was to collect only tweets from particulars where the gender could be clearly determined, as explained in the next section.

### Participant Selection Process

We modeled our data collection methods on prior studies that have used the Twitter platform for generating a convenience sample of users with publicly available accounts, who self-identify as stroke survivors in their profile or tweets.

We then confirmed the self-reported stroke diagnosis by having one researcher generate this initial list of Twitter users and a second researcher check the details for each Twitter user on the list to ensure correct identification of stroke survivors users.

We then employed a stepwise process for coding each Twitter user’s gender as male, female, or unknown/insufficient data. Two researchers independently used these codes, beginning with each Twitter user’s username, followed by profile name, profile description, profile photo, and tweets. Both researchers then reviewed their final gender codes for each Twitter user to ensure consistency and resolve disagreements.

### Data Cleaning

The final sample was prepared for analysis by using the *quanteda* R package. This included the process of basic normalization (eg, remove punctuation and lowercase all text), stop word removal (eg, the words “a” and “the”), normalization of Twitter user mentions (eg, “@janedoe” is converted to “@user”), lemmatization (eg, “dog,” “dogs,” and “dog’s” are all converted to “dog”), and nonprintable character removal (eg, emojis). All analyses relied on public, anonymized data; adhered to the terms and conditions, terms of use, and privacy policies of Twitter; and were performed under Institutional Review Board approval from the authors’ institution.

We do not report any specific tweets that could be used to identify the original Twitter user who posted the content online, as this is an important concern that has been discussed extensively in recent literature on the ethics of using Twitter data for research [29].

### Sentiment Analysis

We calculated the overall frequencies of emotion words for each Plutchik category for each user (and therefore gender) by using the *syuzhet* R package [30]. The NRC Word-Emotion Association Lexicon is available via open access and has been implemented in the get_nrc_sentiment() function of the *syuzhet* R package. Finally, the data were subjected to statistical analyses: For each tweet, given an emotion X, an emotion proportion score was calculated as:

*proportion_X_ = frequency of words with emotion X in a tweet / (frequency of negative words in a tweet + frequency of positive words in a tweet) (equation 1)*

The emotion proportion scores for men and women were then subjected to a Wilcoxon rank sum test in R, since the *F*-test had revealed that the two distributions did not meet the criterion of variance homogeneity [31].

### Structural Topic Models

Considering the final sample of tweets from the data cleaning phase presented above as the starting point, we proceeded as follows:

1. Convert cleaned tweets to tm corpus and create a term document matrix (TDM) using the *tm* R Package [32].
2. Calculate the term frequency inverse document frequency (TF-IDF) for all the words in TDM.
3. Exclude all the words with TF-IDF≤0.1 to remove all the words that are less frequent.
4. Calculate the optimal number of topics (K) in the corpus using the log-likelihood method for the calculated TDM using Gibbs sampling and exploring different metrics: “Griffiths2004,” “CaoJuan2009,” “Arun2010,” and “Deveaud2014” using the *FindTopicsNumber* function from the *ldatuning* R package [33].
5. Apply the spectral method using the *stm* package to discover topics.
6. Topic validation (semantic coherence and exclusivity).
7. Visualization and interpretation of results from the calculated model.

A unique feature of STM, implemented by the *stm* R package [34], is that it can model how the document level covariates affect the topical prevalence parameter μ with a generalized linear model. As mentioned in the Sentiment Analysis section above, our covariate is the gender factor with two levels (“Woman” and “Man”).

Besides the inclusion of the gender covariate, the *stm* R package supports the explicit estimation of correlations among topics. This feature provides further information on the corpus structure. Correlations are estimated by replacing the Dirichlet distribution in the standard LDA framework with a logistic normal distribution as in the Correlated Topic Model [35].

This allows us to identify when two topics are likely to cooccur within tweets (here, we focus on both positive and negative correlations, which are also useful to identify gender differences).

### Hedonometer

We applied the hedonometer tool to all tweets and to the main identified topics as follows: For each word in each tweet, we obtained a happiness score, calculated the mean happiness score for each day, and plotted it by date grouping by gender; STM allows us to identify the main topics and label the topics as “More likely Women” and “More likely Men.” As each topic is defined by a set of words, we obtained the happiness score of each word using the hedonometer, and therefore, we are able to compare topics according to their happiness score. This also allows us to select, for example, the top 25 words with the highest levels of happiness and identify if such words belong to female or male topics.

## Results

### Sample Description

After the selection process, a final sample of 479 Twitter users who posted 800,424 tweets between August 1, 2007, and December 1, 2018, were selected. Women (n=244) posted a total of 3,788,069 tweets; from them, we collected 396,898 tweets (the most recent ones, up to December 2018), and the mean number of tweets posted by our selected sample was 1620. In addition, 54% of the selected sample posted more than 1000 tweets and 71% posted more than 500 tweets. The total number of followers of the selected sample was 182,807.

Men (n=235) posted a total of 1,469,364 tweets, from which we collected 403,526 tweets (the most recent ones, up to December 2018), and the mean number of tweets posted by our selected sample was 1717. In addition, 59% of the selected sample posted more than 1000 tweets and 73% posted more than 500 tweets. The total number of followers of the selected sample was 255,053.

**Figure 1:**
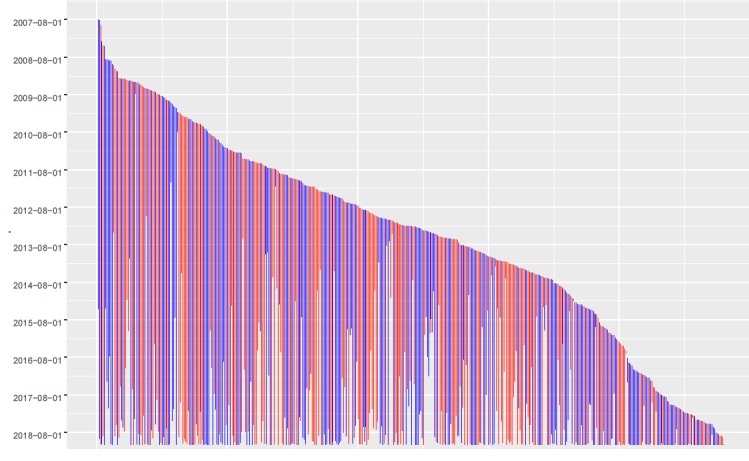
Topics and gender covariate obtained with spectral structural topic modeling.

Figure 1 shows the date of the first and last posted tweets for each selected participant included in our sample (women in red, men in blue; same code colors throughout the analysis). Each vertical line in the plot represents a participant whose first tweet was posted at the top of the vertical line and last tweet was posted at the bottom of it. We ordered participants in the plot from left to right, where the earliest date of the first tweet is shown leftmost for each participant. For example, the leftmost participant is a man whose first tweet was posted in 2007 and last tweet was posted in 2014.

In Multimedia Appendix 1, we present the number of tweets posted by year; a larger number of tweets was posted in 2018 (about 300,000) and the other 500,000 posts were distributed with growing tendency since 2007, as presented in previous research (described in the #Stroke section).

For each of the 479 participants, we reviewed their profiles to verify their geographic locations, obtained by means of the *rtweet* library. We were able to identify the geographic locations of 378 of the 479 users (78.91%).

In Multimedia Appendix 1, we present the total number of users (N=378) by country, showing that most of the users are from four countries: 95% are from Australia, Canada, the United Kingdom, or the United States.

United States had the most users (206/378; 55%). The United Kingdom had 113 users (29.89%). As such, both countries together accounted for more than 85% of the participants.

In Multimedia Appendix 1, we present wordclouds of the top 500 words in all participants’ profile description. Most words are repeated in both wordclouds, but some distinctive characteristics can be observed (women clearly refer to Music, Live, and Time, while men do not).

### Sentiment Analysis

The NRC Word-Emotion Association Lexicon, which contains 10,170 lexical items that are coded for Plutchik’s basic human emotions [23] and implemented in the *syuzhet* R package, associates an emotion (or more than one emotion) to each of the 10,170 lexical items. Given a word and emotion X, the NRC Word-Emotion Association Lexicon associates a score (range: 0 to 1) with it. A score of 1 indicates that the word conveys the highest amount of emotion X. A score of 0 indicates that the word conveys the lowest amount of emotion X.

We then identified (via the get_nrc_sentiment() function) the number of words that, according to the NRC, express positive or negative sentiment as well as one (or more than one) of Plutchik’s eight basic emotions.

Table 1 summarizes the raw number of words (and their percentages) obtained with the get_nrc_sentiment() function of the *syuzhet* R package.

**Figure 2:**
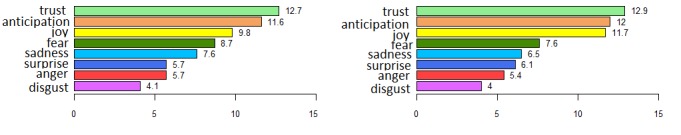
Ranking of emotions (in percentage of the total words. Men: left; women: right). Each bar represents the percentage of total words presented in Table 1.

Among both men and women, the most frequent emotions were trust, anticipation, and joy (top 3), as shown in Figure 2.

Women used considerably more words from all positive categories (except anger), and men used more words in all negative categories (except surprise), as shown in Table 1.

When considering negative or positive words, women used 12% of negative words, while men used 13.6% of negative words. In contrast, women used 21.8% of positive words, while men used 20.5% of positive words. Positive and negative labels for words are also obtained from the NRC lexicon using the get_nrc_sentiment() function of the *syuzhet* R package.

**Table 1:** Raw frequencies of words identified for each emotion.

Emotion	Men, n (%)	Women, n (%)
Anger	76,650 (5.7)	74,858 (5.4)
Anticipation	155,608 (11.6)	166,150 (12.0)
Disgust	55,512 (4.1)	54,785 (4.0)
Fear	117,221 (8.7)	104,826 (7.6)
Joy	131,243 (9.8)	161,933 (11.7)
Sadness	101,475 (7.6)	89,868 (6.5)
Surprise	77,109 (5.7)	83,663 (6.1)
Trust	170,0176 (12.7)	178,718 (12.9)
Negative	182,288 (13.6)	166,000 (12.0)
Positive	276,124 (20.5)	300,751 (21.8)

^a^Not applicable because .

^b^N/A: not applicable.

We then calculated the emotion proportion score for each emotion X, as shown in equation 1 in the Methods section.

Table 2 reports statistical comparisons; for example, for the global positive emotion, women (median=100%, mean=65.57%) used considerably more positive words than men (median=66.67%, mean=60.73%). Since the *F*-test indicated that the two distributions have a significantly different variance (*F*_237040,242190_=1.0468, *P*<.001), they were subjected to a Wilcoxon rank sum test. This test showed that the difference between men and women is highly statistically significant (W=2.6817e+10, *P*<.001). Similar results are shown in Table 2 for global negative emotion: Men used considerably more negative words than women; in addition, each individual positive emotion (joy, anticipation, and trust, except surprise) was favorable to women and each individual negative emotion (fear, sadness, and disgust) was preferred by men.

**Figure 3:**
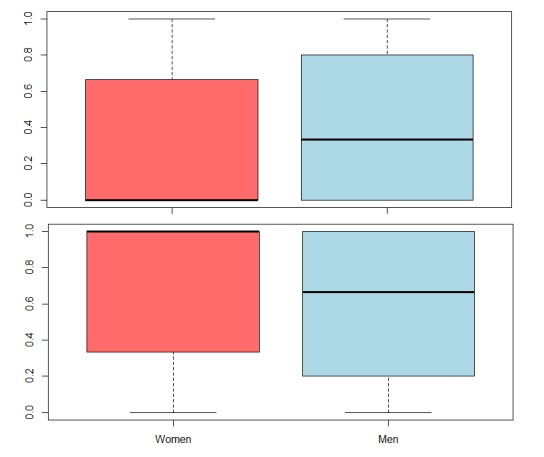
Topics and gender covariate obtained with spectral structural topic modeling.

Global negative-positive proportion comparisons are presented in Figure 3. Women used considerably fewer negative words and more positive words than men (shown at the top and bottom of Figure 3, respectively)

Plutchik’s eight emotions are subdivided into four complementary pairs, namely, joy–sadness, anticipation–surprise, trust–disgust, and anger–fear [23].

**Table 2:** Statistical comparison of words identified for each emotion.

Emotion, participants		Median	Mean	*F (df)*	*P* value	W	*P* value
**Joy**				0.9030 (237040,242190)	<.001	2.63e+10	<.001
	Men	0	0.2972	—	—	—	—
	Women	0	0.3611	—	—	—	—
**Negative**				1.0468 (237040,242190)	<.001	3.05e+10	<.001
	Men	0.3333	0.3927	—	—	—	—
	Women	0	0.3443	—	—	—	—
**Fear**				1.1183 (237040,242190)	<.001	3.01e+10	<.001
	Men	0	0.2481	—	—	—	—
	Women	0	0.2121	—	—	—	—
**Positive**				1.0468 (237040,242190)	<.001	2.68e+10	<.001
	Men	0.6667	0.6073	—	—	—	—
	Women	1.0000	0.6557	—	—	—	—
**Sadness**				1.0261 (237040,242190)	<.001	2.45e+10	<.001
	Men	0	0.2204	—	—	—	—
	Women	0	0.1868	—	—	—	—
**Anger**				1.0399 (237040,242190)	<.001	2.90e+10	<.001
	Men	0	0.1573	—	—	—	—
	Women	0	0.1495	—	—	—	—
**Anticipation**				0.9837 (237040,242190)	<.001	2.79e+10	<.001
	Men	0	0.3299	—	—	—	—
	Women	0	0.3488	—	—	—	—
**Surprise**				1 (237040,242190)	>.99	N/A^b^	N/A
	Men	0	0.1672	—	—	—	—
	Women	0	0.1779	—	—	—	—
**Trust**				1.0134 (237040,242190)	<.001	2.81e+10	<.001
	Men	0.1667	0.3628	—	—	—	—
	Women	0.2500	0.3755	—	—	—	—
**Disgust**				1.0559 (237040,242190)	<.001	2.88e+10	<.001
	Men	0	0.1153	—	—	—	—
	Women	0	0.1109	—	—	—	—

**Figure 4:**
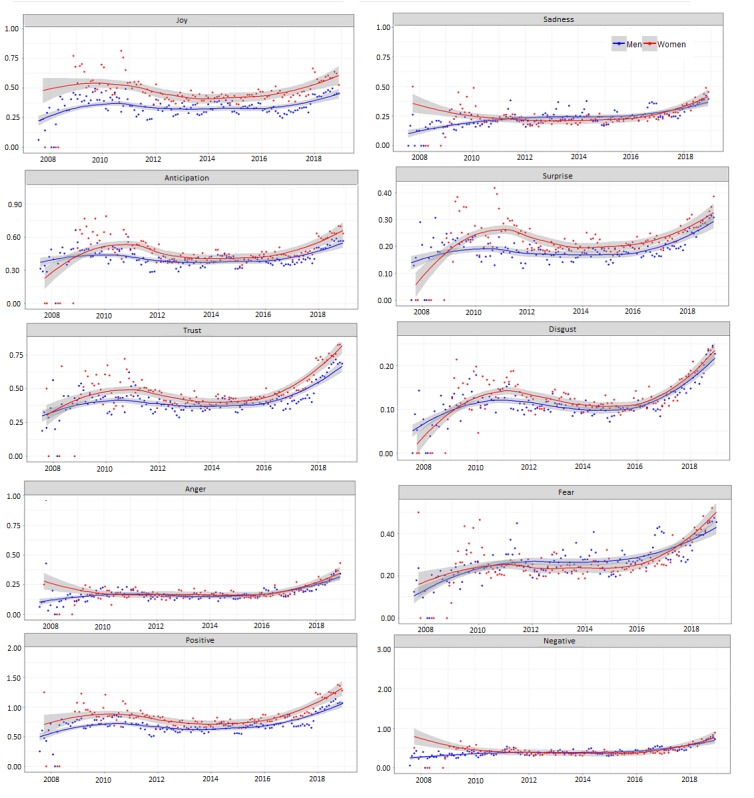
Monthly mean scores for NRC emotions in the 2008-2018 period.

Figure 4 plots such scores for each emotion summarized monthly along all the time periods in the study of emotion words for each pair of emotions, obtained with *syuzhet* R package and plotted with the *ggplot2* R package. It clearly shows higher scores for women in positive emotions along time and lower scores for men in almost every emotions throughout the considered period.

As shown in Figure 4, joy and global positive words clearly present higher values for women throughout the considered period.

### Assigning Happiness Scores With the Hedonometer

We then calculated the happiness score using the hedonometer for each word in each tweet, summarized the mean happiness score for each day during the whole period under study, and plotted it by date, grouping by gender.

**Figure 5:**
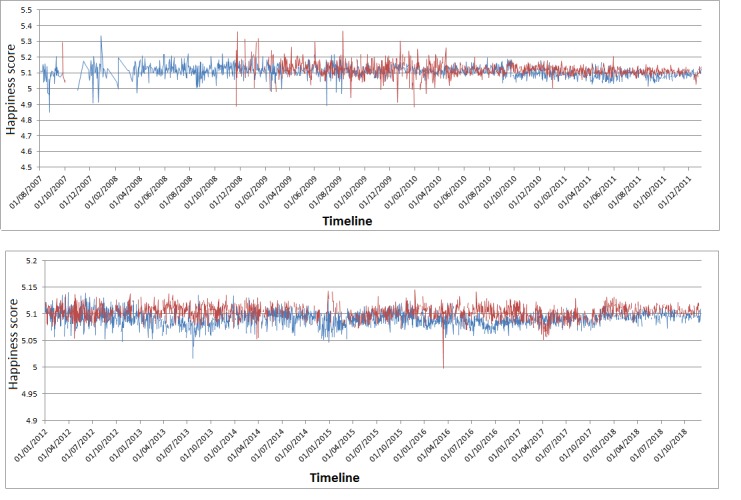
Topics and gender covariate obtained with spectral structural topic modeling.

As shown in Figure 5, happiness ratings obtained by hedonometer summarized on a daily basis for each user are also higher for women than for men, almost throughout the considered time period, with remarkable differences in favor of women, for example, in the 2013-2014 period, 2016, and 2018.

### Structured Topic Modelling

Before the application of STM, we performed an exploratory cluster analysis using VosViewer [36]. As defined by VosViewer, a term map is a two-dimensional representation, in which strongly related terms are located close to each other and less strongly related terms are located further away from each other. Each point in a term map has a color that depends on the density of items at that point. It is argued that the VOS mapping technique yields more satisfactory term maps than popular multidimensional scaling–based approaches to bibliometric mapping. Maps constructed using these multidimensional scaling–based approaches are shown to suffer from certain artifacts. Maps constructed using the VOS mapping technique do not have this problem, as reported by Waltman et al [36]. Details are presented in Multimedia Appendix 1 (VosViewer Cluster Analysis).

We tested different parameter configurations to increase intercluster distances and reduce intracluster distances. VosViewer allowed us to identify seven clusters for men and five clusters for women (Multimedia Appendix 1). In the obtained clusters for the most relevant 250 words for men and women, we highlighted words that are common to clusters obtained by men and women. Unfortunately, this is the case for most of the words; therefore, it did not allow us to visually identify gender differences.

Nevertheless, in Multimedia Appendix 1, we present the clusters for the words that are not common to both men and women, and we applied the hedonometer to each of them; the happiness scores are shown in brackets for each word.

We summarized the happiness scores and obtained mean happiness scores of 5.31 (SD 1.31) for all the words present only in men clusters and 6.25 (SD 1.07) for those present only in women clusters. We then considered each of the largest clusters separately and obtained the following for men: mean happiness score in cluster 1=5.52 (SD 0.99) and mean happiness score in cluster 2=5.06 (SD 1.25). For women, the mean happiness score was 6.30 (SD 1.12) in cluster 1, 5.70 (SD 1.13) in cluster 2, and 6.68 (SD 0.81) in cluster 3. Again, happiness scores of women were higher than those of men when considering the scores at the cluster level.

Before STM, we also performed LDA analysis for seven topics; the number of topics was determined as shown in Multimedia Appendix 1 by using different metrics and the FindTopicsNumber function from the *ldatuning* R package.

The obtained topics are presented in Multimedia Appendix 1, but as with cluster analysis, we could not identify topics clearly related to men or women.

**Figure 6:**
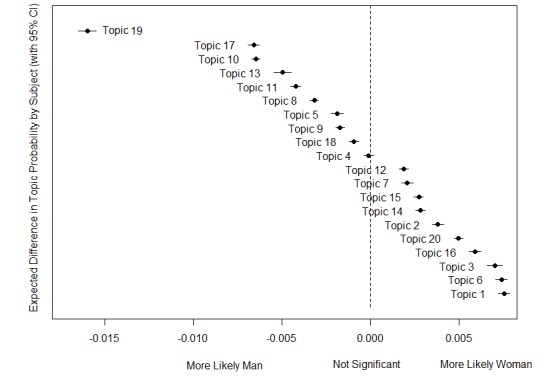
Topics and gender covariate obtained with spectral structural topic modeling.

Therefore, we applied STM to associate covariates (Gender) to the identified topics and plot results as presented in Figure 6. As with most topic models, the objective function maximized by STM is multimodal. Therefore, the way we choose the starting values for the variational EM algorithm can affect our final solution.

We applied LDA initialization (the default option), which uses several passes of collapsed Gibbs sampling to initialize the algorithm.

Table 3 shows the top three topics selected for men and women, with 3 different types of word profiles: highest probability, frequency-exclusivity (FREX), and score values. Detailed descriptions of all identified topics are presented in Multimedia Appendix 1.

FREX measures exclusivity of the words to the topic in a way that balances it with word frequency. The score metric divides the log frequency of the word in the topic by the log frequency of the word in other topics, and highest probability considers words within each topic with the highest probability, inferred directly from topic-word distribution.

In Multimedia Appendix 1, we present the evaluation of the obtained topics. Semantic coherence is a criterion developed by Mimno and colleagues [37]; it is maximized when the most probable words in a given topic frequently cooccur together, and it has been shown that the semantic coherence metric correlates well with human judgment of topic quality [37].

Topics 19, 17, and 10 show all semantic coherence values to the right side of the plot, with topic 19 to the rightmost possible position and close to topic 10. Topics 1, 6, and 3 are in the central positions, while upper right side positions are six optimal selected topics showing acceptable values.

We then assigned happiness scores to topics with the hedonometer tool (we proceeded as was done with VosViewer clusters described in Multimedia Appendix 1). As shown in Figure 6, STM allows us to label the topics as “More likely Women” and “More likely Men.”

As presented in Table 3, each topic is defined as a list of 20 words. To assign happiness scores, we selected the words with the highest probability (first row of each topic in Table 3). Therefore, we applied the hedonometer tool to assign a happiness score to each word with the highest probability of each topic.

In the Multimedia Appendix 1, we present the complete list of all words defining each topic. For each word, we present the happiness score and its corresponding topic (and therefore the associated gender to the topic). We selected a subset of these words (Tables 4 and 5). We show the 25 words with the highest happiness scores and those with the lowest happiness scores along with the corresponding gender.

Table 4 shows that 19 of the 25 words with the highest happiness scores correspond to women’s topics and only 6 correspond to men’s topics. Table 5 shows the 25 words with the lowest happiness scores, and only 7 corresponded to women, while 18 corresponded to men.

**Table 3:** Top three identified topics and percentages for women (topics 1, 3,6) and men (topics 10,17,19).

Topic (%)	Highest probability	FREX^a^	Score
1 (5.23)	year, happy, tomorrow, open, birthday, take, come, busy, christmas, baby, sleep, friday, sunday, monday, list, bed, smile, market, treat, guess	merri, birthday, appl, ang, eve, awak, con, clay, decemb, angel, happi, est, syracus, ako, relax, closet, lang, store, carousel	happi, year, birthday, tomorrow, open, christma, sleep, friday, come, busi, babi, sunday, take, bed, store, monday, holiday, list, date, market
3 (7.21)	good, great, video, hope, night, morn, lol, tonight, long, done, head, weekend, fun, readi, celebrate, citi, movie, luck, earli, forget	playlist, chicken, grill, peter, egg, chees, movi, delici, potato, cooki, bbq, soup, recip, video, kitti, cup, chili, luck, pan, belli	good, video, hope, night, morn, great, lol, weekend, playlist, movi, tonight, don, luck, fun, long, sweet, forget, gonna, saturday, dinner
6 (5.73)	time, life, thing, world, god, famili, twitter, hear, power, hate, pass, speak, human, step, posit, bless, super, continu, messag, creat	god, lord, pray, faith, amen, bless, prayer, psalm, soul, negat, holi, heal, thank, charl, nchousingbuild, merci, evil, accomplish, yea, compass	time, god, thing, life, famili, twitter, world, lord, bless, hear, power, step, super, hate, pray, prayer, congrat, pop, faith, posit
10 (3.82)	end, heart, walk, news, stop, run, hand, pay, mile, rate, worth, success, dead, offer, singl, reach, staff, fail, snow, hero	mile, rate, bpm, attitud, anthem, bioness, hawk, failur, shoulder, flaw, casual, complic, tattoo, zombi, hero, pinterest, hand, virus, vancouv	heart, walk, end, news, mile, stop, run, rate, bpm, hand, pay, dead, success, attitud, hero, snow, worth, bbc, offer, reach
17 (5.25)	back, play, game, team, job, place, boy, man, point, won, lost, black, park, perfect, act, lose, john, footbal, film, player	yard, hole, playoff, player, joe, nfl, eagl, cowboy, bronx, kiss, dalla, theater, doodl, lewi, cunt, throw, golden, barn, korea, brave	game, team, play, back, boy, job, footbal, player, park, perfect, black, act, place, north, beat, test, film, lose, tour, kick
19 (6.25)	stroke, support, find, survivor, learn, lot, brain, care, health, patient, help, aware, money, raise, children, research, import, risk, experience, hospital	aware, raise, foundat, risk, research, patient, region, medic, lot, recoveri, donat, increas, factor, studi, resourc, rehab, treatment, cancer, rehabilit, learn	stroke, survivor, learn, lot, find, support, brain, patient, awar, care, rais, health, research, risk, foundat, injuri, studi, region, recoveri, disease

^a^FREX: frequency-exclusivity.

**Table 4:** Top 25 words with highest happiness scores, topics, and gender of participants.

Word	Participant	Score	Topic
Love	Women	8.42	T16
Happy	Women	8.3	T1
Win	Women	8.12	T15
Smile	Women	8.1	T1
Won	Men	8.1	T17
Music	Women	8.02	T2
Weekend	Women	8.0	T3
Celebrate	Women	7.98	T3
Christmas	Women	7.96	T1
Fun	Women	7.96	T3
Free	Men	7.96	T11
Great	Women	7.88	T3
Success	Men	7.86	T10
Award	Women	7.86	T15
Positive	Women	7.8	T6
Hero	Men	7.8	T10
Sun	Men	7.8	T11
Birthday	Women	7.78	T1
Winner	Women	7.78	T15
Beauty	Men	7.76	T5
Family	Women	7.72	T6
Gift	Women	7.72	T15
Brilliant	Women	7.68	T2
Super	Women	7.68	T6
Amazing	Women	7.66	T16

**Table 5:** Top 25 words with the lowest happiness scores, topics, and gender of participants.

Word	Participant	Score	Topic
Death	Men	1.54	T18
Kill	Women	1.56	T12
Die	Men	1.74	T18
Fail	Men	1.96	T10
Dead	Men	2.0	T10
Pain	Men	2.1	T4
Hell	Men	2.22	T9
Poor	Men	2.32	T9
Hate	Women	2.34	T6
Sad	Women	2.38	T12
Attack	Men	2.42	T8
Shot	Women	2.5	T2
Shit	Men	2.5	T18
Aphasia	Men	2.58	T11
Stroke	Men	2.58	T19
Lie	Men	2.6	T13
Bad	Women	2.64	T16
Fight	Women	2.7	T16
Lost	Men	2.76	T17
Lose	Men	2.76	T17
Disabled	Men	2.82	T18
Problem	Men	2.98	T4
Wrong	Men	3.14	T18
Forget	Women	3.22	T3
Cut	Men	3.42	T9

**Figure 7:**
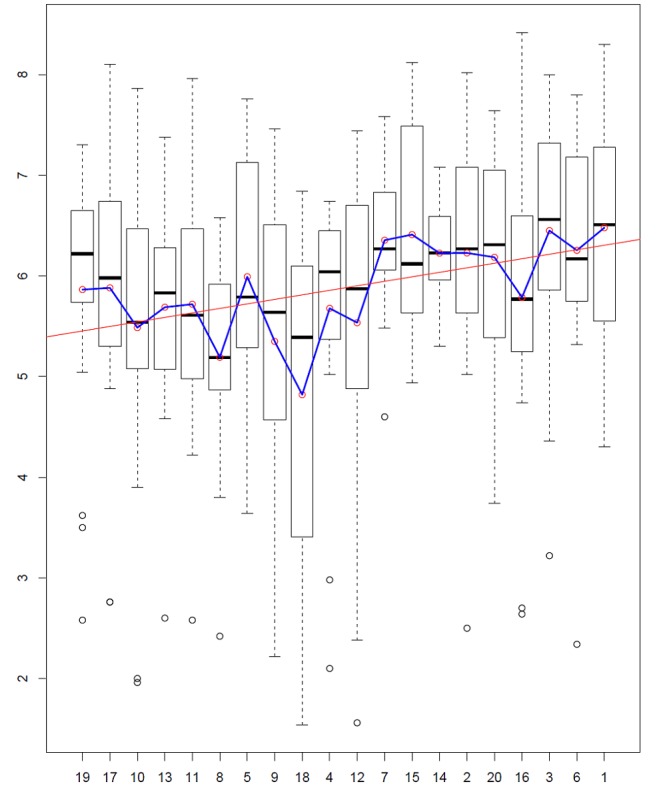
Topics and gender covariate obtained with spectral structural topic modeling.

We then calculated the boxplots of the happiness scores for each topic (Figure 7), ordered from “More Likely Men” to “More Likely Women”; the means and regression line are shown in red circles and a red line, respectively (*P*<.001).

Figure 7 shows higher happiness scores from Topic 4 to the right (ie, women’s topics) with the exception of Topic 16, which contains several words with low happiness scores (eg, “bad” or “lone”; Multimedia Appendix 1). The regression line shows a positive slope in the direction of women’s topics (*P*<.001).

**Figure 8:**
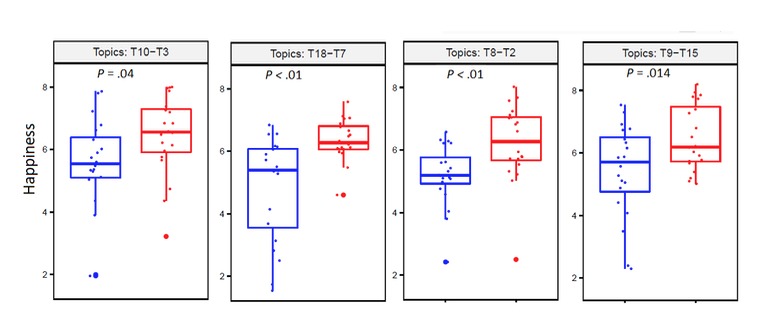
Topics and gender covariate obtained with spectral structural topic modeling.

We then compared happiness scores by pairs from the leftmost and rightmost topics in Figure 6 to the center (Topic 19-Topic 1, Topic 17-Topic 6, Topic 10-Topic 3, etc). We found significant differences in favor of women in 4 of the 10 pairs of topics (and none in favor of men) when comparing the happiness scores by pairs of topics (Figure 8; men blue, women red). The complete list of comparisons is presented in Multimedia Appendix 1.

**Figure 9:**
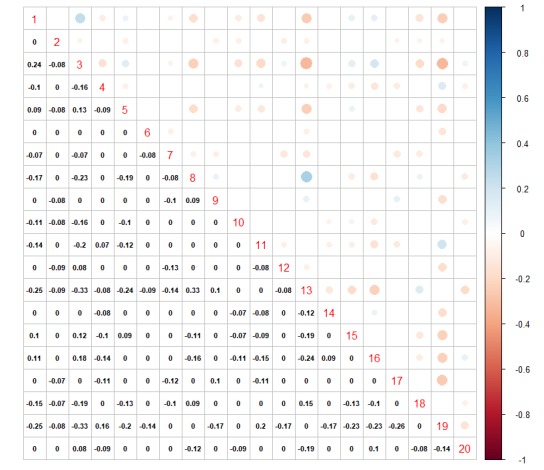
Topics and gender covariate obtained with spectral structural topic modeling.

STM also permits correlations between topics. Positive correlations indicate that both topics are likely to be discussed in a tweet. In Figure 9, we plot both positive and negative correlations for all identified topics.

Topic 1 shows the highest positive correlation with Topic 3. This can be further confirmed in Table 3, as both topics address actual positive everyday life situations like celebrations (birthday, Christmas, holiday, merry), and Topic 1 was strongly negatively correlated with Topic 19, which refers to research, studies, risks, factors, hospital, disease, stroke, and care.

Topic 3, therefore, is also strongly negatively correlated with Topic 19 and Topic 10.

Topic 10 refers to running, beats per minute, heart rate, attitude, stop, walk, and reach, while Topic 3 refers to fun, celebrate, movie, Saturday, dinner, barbeque, chicken, grill, egg, cheese, delicious, potato, and cooking. Topic 6 addresses religion—god, lord, pray, faith, amen, bless, prayer, psalm, soul—while Topic 17 addresses sports—playoff, nfl, game, yard, football, player—showing clear differences in topics of interest addressed by men and women.

## Discussion

### Principal Findings

In this work, we proposed the application of Twitter lists to a chronic health condition in a GNU framework (R-3.5.1). We applied a set of publicly available R libraries for collecting and organizing Twitter data via Twitter’s REST and stream API (*rtweet*), sentiment analysis (*syuzhet*), text mining (*tm, quanteda*), and structural topic model (*stm*). We also applied the hedonometer tool to assign happiness scores to topics by gender.

According to our findings, men use significantly more words while expressing negative emotions in their tweets than women, while women use significantly more words when expressing positive emotions.

The results also show that the top three most frequent emotions evoked by both men and women are trust, anticipation, and joy. Besides, the statistical analysis of the basic emotions detects significant preferences for each gender: While words from the emotional fields of trust, anticipation, and joy appear significantly more often in women’s tweets, men’s tweets significantly exhibit a preference for evoking disgust, anger, fear, and sadness.

We also applied another tool that was specifically designed for considering Twitter inputs—the hedonometer. Happiness ratings obtained by the hedonometer, summarized on a daily basis for each user, are also higher for women than for men almost all along the considered time period.

Finally, we applied structural topic modelling (to the best of our knowledge, for the first time to a chronic health condition) to identify main topics addressed by gender and determined positive and negative correlations between topics by gender.

Topics in this context are defined as sets of words; therefore, we assigned happiness scores to the words with highest probabilities in the identified topics and found that the topics women talk about show higher happiness scores than the topics addressed by men.

A common stereotype in both Western and Eastern cultures suggests that women are more emotional than men, particularly when responding to negative emotions [38]. As remarked in the Introduction section, after stroke, women experience more activity limitations, worse health-related quality of life, and more poststroke depression than men [7] and are twice as likely to suffer from severe depression following a stroke than men. We identify several explanations for our findings, listed below.

First, according to Ayis et al [8], women draw larger components of their sense of self and self-worth from interpersonal relationships and networks, and they are more sensitive to adversities of these. Therefore, female stroke survivors may experience (to a larger extent in comparison to men) the interpersonal and intrapersonal benefits of sharing positive events and emotions on social network sites (SNSs). The intrapersonal benefits of sharing positive events and emotions on SNSs consist of re-experiencing and prolonging these positive events; the interpersonal benefits comprise positive social interaction and positive feedback from other SNS users (according to the results of an ethnographic diary study on Facebook use from Sas et al [39]).

Second, prior research indicates that the positivity of self-presentation on SNSs has an influence on both the quantity and quality of reactions from other SNS contacts. For example, Utz [40] found that SNS users were least likely to receive reactions from their online friends when they expressed sadness in their postings. Similarly, Forest and Wood [41] demonstrated that more positive status updates on Facebook received more positive and favorable feedback from friends than negative status updates.

A third explanation to our findings can be related to the existence of “the positivity bias in SNS communication,” which states that “while the SNS environment generally enables authentic self-presentation, it favors positive forms of authenticity over the presentation of negative aspects of the true self” [42].

Therefore, according to Reinecke et al, due to the positivity bias in SNS communication, individuals with higher levels of psychological well-being have a higher chance of experiencing authenticity through the use of SNSs than SNS users with low psychological well-being.

The fourth explanation is related to a recent Facebook analysis involving 15,000 users [43]. The authors concluded that “language used more by self-identified females was interpersonally warmer, more compassionate, polite, and—contrary to previous findings—slightly more assertive in their language use, whereas language used more by self-identified males was colder, more hostile, and impersonal.” In fact, the following text from their publication, can also be applied to our own findings:

The most strongly female-linked topics included words describing positive emotions (e.g., “excited”, “happy”, “<3”, “love”,), social relationships (e.g., “friends”, “family”, “sister”), and intensive adverbs (e.g., “sooo”, “sooooo”, “ridiculously”). Strongly male-linked topics included words related to politics (e.g., “government”, “tax”, “political”), sports and competition (e.g.,“football”, “season”, “win”, “battle”), and specific interests or activities, such as shooting guns, playing musical instruments, or playing video games.

Therefore, according to this fourth explanation, our findings in another SNS such as Twitter are similar to those involving users not necessarily identified as stroke survivors on Facebook.

### Limitations

The collected sample was not intended to be representative or a comprehensive set of all tweets posted by stroke survivors during the period under study. Although the collected data also included tweets directed at other users (ie, conversational tweets), the results cannot be considered to reflect all topics of conversation appearing in Twitter for stroke survivors.

Data collection relied on Twitter’s streaming API, which prevents collection of tweets from private Twitter accounts. As a result, findings may not represent individuals with private accounts.

Furthermore, recent analysis [44] shows that 62% of all Twitter users are less than 49 years old; our participants are skewed toward such an age range, and most of them from the United States.

Nevertheless, as discussed in the Introduction section, multiple recent studies have reported a sustained increasing incidence of stroke at younger ages and the included participants were randomly selected after checking their membership to several Twitter stroke–related lists and manually double checked in relation to gender and stroke survivor condition.

We analyzed women (n=244) who posted a total of 3,788,069 tweets. From them, we included 396,898 tweets in our analysis (the most recent ones, up to December 2018); therefore, we analyzed 10.5% of all posted tweets by women participating in this study.

We analyzed men (n=235) who posted a total of 1,469,364 tweets. From them, we included 403,526 tweets in our analysis (the most recent ones, up to December 2018). Therefore, we analyzed 27.4% of all posted tweets by men participating in this study.

The total number of tweets posted by women from whom we extracted our sample is clearly larger than tweets posted by men. This seems to be coincidental with general Twitter use statistics: Women are usually more active, and each month, 40 million more women than men visit Twitter [45].

Other relevant factors to be mentioned as limitations to our study are related to geographic location, spatial trajectory, or the time of the day a tweet has been posted. As remarked by Padilla et al [46] and Gore et al [47], such factors may affect tweets’ sentiments. We observed that 85% of our participants profiles are from the United Kingdom and United States, but spatiotemporal aspects are not controlled in our study.

Finally, the individual psychological differences that stroke survivors may experience must also be mentioned. Certain individuals might have personality traits that make them more predisposed to positive or negative sentiments. The degree to which sentiment reflects variance in psychological traits versus the situational context in which those traits were expressed is unclear. Possible users affected by severe depression may not be active on Twitter; this could be a source for another significant bias in the data sample.

### Comparison with Prior Work

One of the scarce previous research about tweet topics or sentiment analysis on chronic health conditions was recently conducted by Brunner and colleagues [48]. Tweets tagged with traumatic brain injury (TBI)-related hashtags were harvested over a one-month period in 2016 and analyzed qualitatively and quantitatively. A total of 29,199 tweets included tweets sent by 893 users, 219 of whom had a brain injury. Twitter was used to discuss health issues, raise awareness of TBI, talk about life after TBI, talk about sport and concussion, and communicate inspirational messages.

In relation to depression, Lachmar and colleagues [49] captured 3225 original tweets for the hashtag #MyDepressionLooksLike that circulated in May 2016. Cleaning resulted in a total of 1978 tweets. Using qualitative content analysis revealed seven themes: dysfunctional thoughts, lifestyle challenges, social struggles, hiding behind a mask, apathy and sadness, suicidal thoughts and behaviors, and seeking relief. Contrary to Lachmar and colleagues' [49] analysis or the #Stroke analysis (the one presented in the Introduction section), our analysis is not linked to a specific hashtag.

It is important to remark the need for further research from a gender perspective, as promoted by initiatives such as the Women’s Brain Project [50].

### Conclusions

This study explored emotional expressivity for eight specific types of emotion and identified 20 main topics of interest through Twitter posts in stroke survivors from a gender perspective. Numerous studies have shown that, compared with men, women usually experience more frequent and stronger negative emotions. Nevertheless, our results show that men present more frequent and stronger negative emotions in their tweets, when considering both globally positive-negative or individual tweets and analyzing them using two different well-established approaches: the Plutchik model and the hedonometer tool.

## Appendix

Multimedia Appendix 1 Demographics, wordclouds, VosViewer cluster analysis, latent Dirichlet allocation topics, correlation analysis, STM topics, hedonometer scores, and the Plutchik psychoevolutionary model.

## Abbreviations

API: application program interface
EM: expectation maximization
FREX: frequency-exclusivity
HRQoL: health-related quality of life
LDA: latent Dirichlet allocation
NRC: National Research Council
REST: representational state transfer
SNS: social network site
STM: structural topic modeling
TDM: term document matrix
TF-IDF: term frequency – inverse document frequency
