# Supplementary material for: Stroke Survivors on Twitter: Sentiment and Topic Analysis From a Gender Perspective
Source: J Med Internet Res. 2019 Aug 26;21(8):e14077. doi: 10.2196/14077 (PMC6732975; doi:10.2196/14077)
Supplement: Multimedia Appendix 1 [file jmir_v21i8e14077_app1.pdf]

Multimedia Appendix

Population description

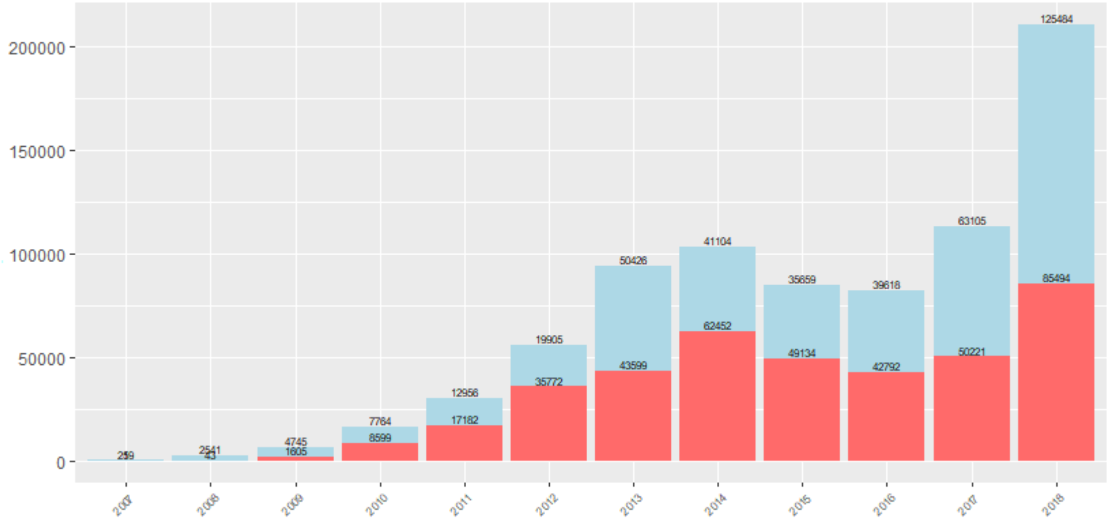

Figure A1. Number of tweets posted by year

Geographic location

For each of the 479 participants we manually reviewed their profiles to verify their geographic locations, obtained by means of rtweet library.

.

We were able to identify the geographic locations of 378 of the 479 users (78.91%). As shown in Table A0, most of the users are from 4 countries, 356 of the 378 users (95%) are from Australia (AU), Canada(CA), United Kingdom (UK) or United States (US).

United States is the country with more users: 206 of the 378, therefore 55% of the users are from United States.

The United Kingdom with 113 users (29.89%) is the second country in representation, therefore both countries provide more than 85% of participants.

Table A0 Geographic location of participants by country

Country ID	Men	Women	Total
AU	11	7	18
CA	9	10	19
CH	1		1
DE	1		1
ES	1		1
FR	3		3
HK	1		1
ID	1		1
IE		1	1
IT	2		2
KE		1	1
MX	1	1	2
MY	2		2
NO	1		1
NZ		1	1
PH	1	1	2
SG		1	1
UK	67	46	113
US	97	109	206
ZA	1		1
Not identified	35	66	101
**Total**	**235**	**244**	**479**

Wordclouds from participants profiles self-description

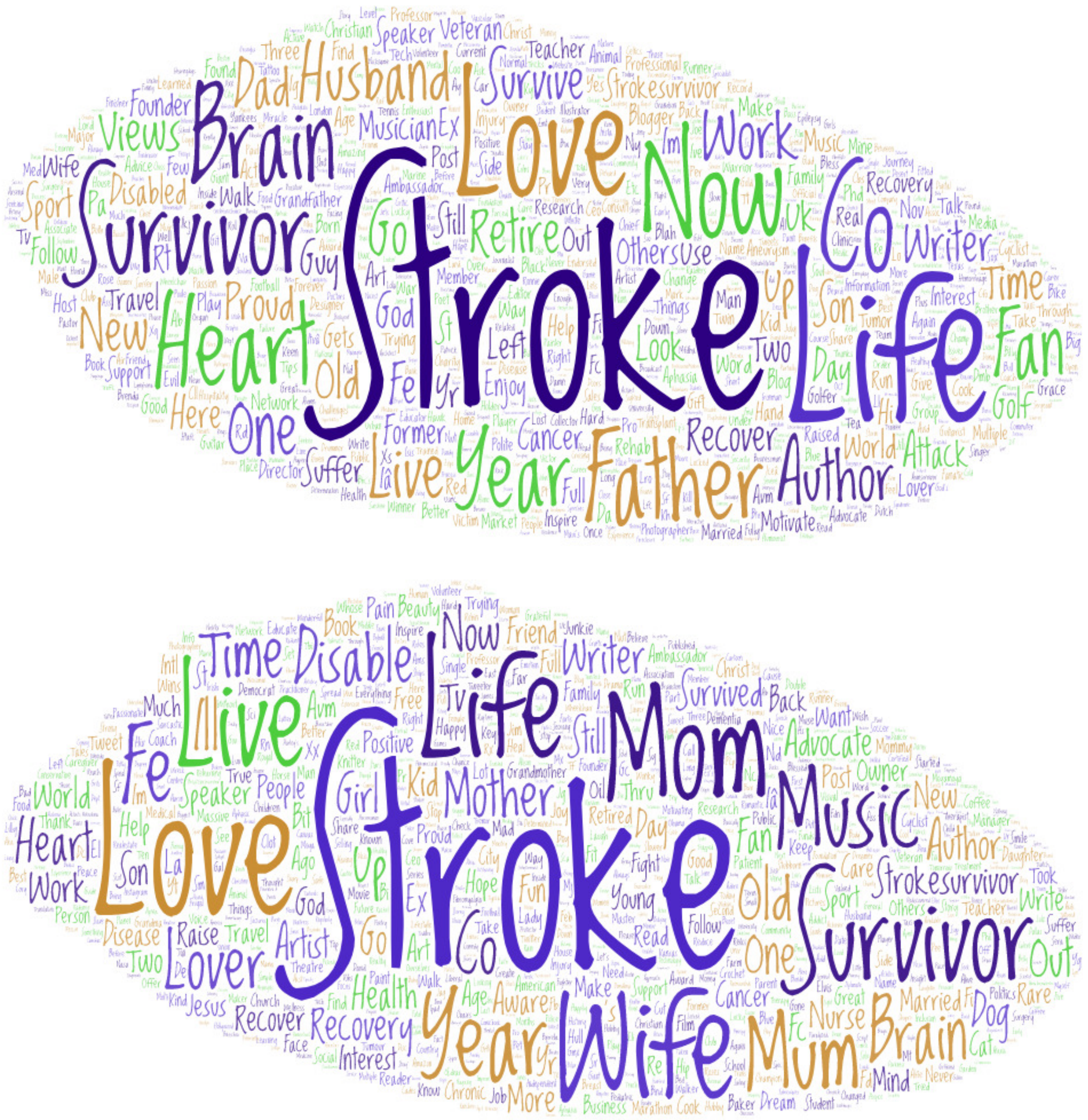

Figure A2. Wordclouds of the top 500 words in users profiles description, men top women bottom

VosViewer Cluster Analysis

As initial exploration of topics analysis, we present a visualization where words that appear together in the text are shown together and words that appear more frequently in the text are highlighted from the others. A term map is a two-dimensional representation of a field in which strongly related terms are located close to each other and less strongly related terms are located further away from each other. While several programs are available for analyzing text units and similarity matrices, the emphasis of VOSviewer is visualization (VOS stands for Visualization of Similarities). It is argued that the VOS mapping technique yields more satisfactory maps than popular multidimensional-scaling-based approaches. Maps constructed using these multidimensional scaling-based approaches are shown to suffer from certain artifacts. Maps constructed using the VOS mapping technique do not have this problem (Waltman et al 2010).

Tweets are tokenized into terms, terms are assigned to clusters by maximizing a quality function. The quality function is a variant of the modularity function of Newman and Girvan (2004) and Newman (2004) developed in the field of network science, based in the Potts model.

In order to maximize the quality function, VosViewer clustering technique uses the smart local moving algorithm introduced by Waltman and Van Eck (2013). The local moving heuristic repeatedly moves individual nodes from one community to another in such a way that each node movement results in a modularity increase. The local moving heuristic iterates over the nodes in a network in a random order. For each node, it is determined whether it is possible to increase modularity by moving the node from its current community to a different (possibly empty) community. If increasing modularity is indeed possible, the node is moved to the community that results in the largest modularity gain. The local moving heuristic keeps moving nodes until a situation is reached in which there are no further possibilities to increase modularity through individual node movements.

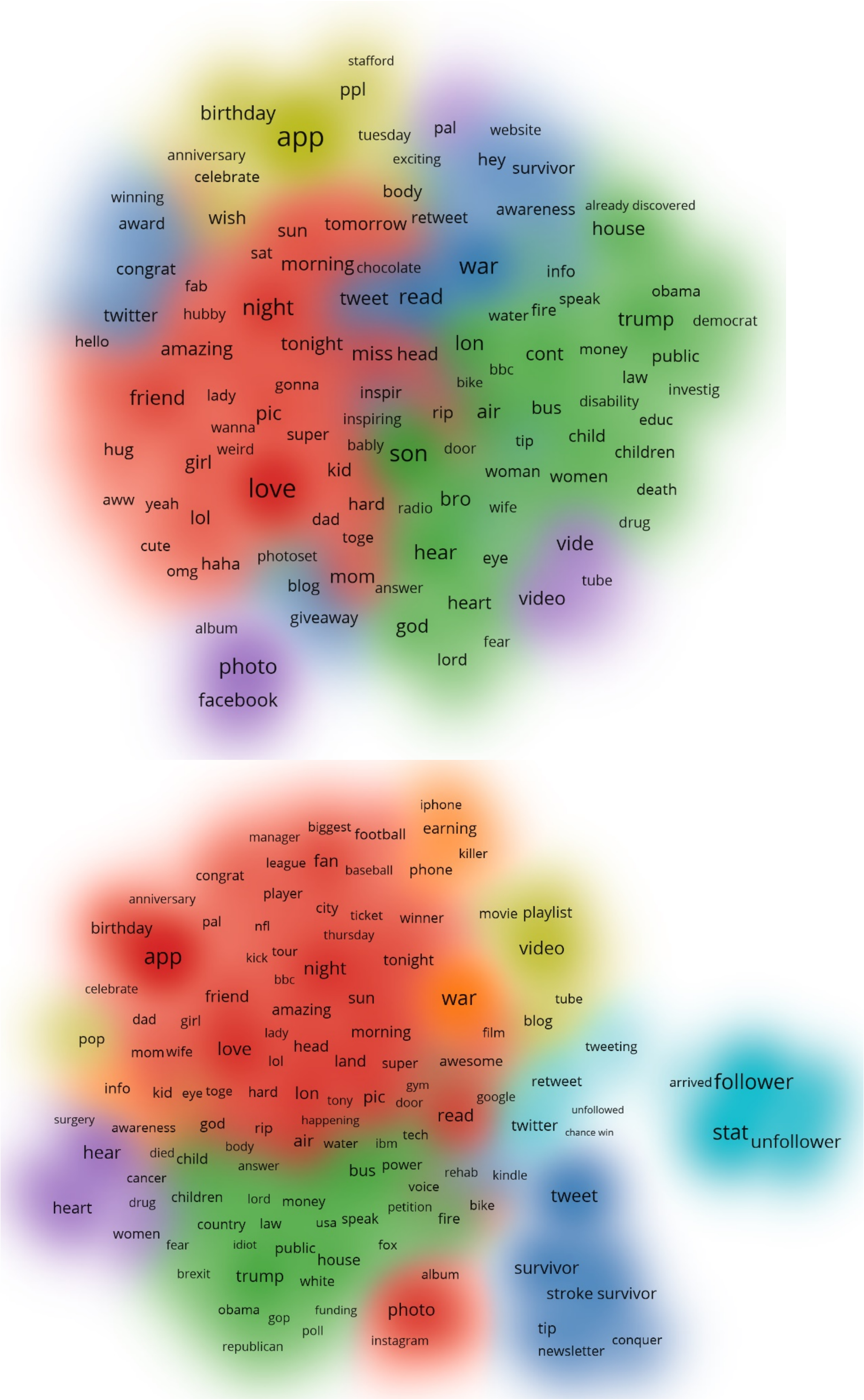

Figure A3. Vosviewer clusters women top, men bottom

Table A1 Tabular representation of Men clusters obtained with VosViewer

**Cluster 1**	league	**rip**	**facebook**	**pic**	**fan**	**award**	**biggest**
	damn	**journey**	**football**	**awesome**	**album**	**pal**	**congrat**
	**giveaway**	**bbc**	brian	**god**	**air**	manager	baseball
	**voice**	**girl**	**announce**	**wife**	**tonight**	**ticket**	**friend**
	**tour**	**kick**	**lady**	couldnt	**amazing**	daily photo	**weekend**
	**massive**	**gonna**	**fantastic**	stadium	gym	**night**	**player**
	**sun**	**photo**	**film**	**morning**	**volunteer**	**thursday**	**tomorrow**
	**app**	**birthday**	nfl	**lol**	**head**	**winning**	manchester
	arsenal	**love**	**episode**	**tough**	**celebrate**	**super**	entry
	happening	**radio**	tony	**lon**	**read**	**dad**	**congratulation**
	**winner**	**died**	**bike**	**toge**	**hard**	**kid**	**city**
	**tuesday**	dude	**body**	sponsor	**mom**	england	**door**
	**anniversary**	land	instagram	**happy birthday**	**gotta**	buddy	
**Cluster 2**	**power**	**speak**	golf	**child**	poll	**obama**	**fear**
	**country**	**white**	petition	**law**	supporter	**bus**	**adult**
	funding	**website**	usa	doubt	**trump**	**brexit**	tech
	**fire**	**launch**	corbyn	**water**	**answer**	wouldn	**disability**
	**money**	**democrat**	conservative	**house**	canadian	clinton	**business**
	**woman**	**veteran**	illegal	**lord**	**congress**	**google**	shouldn
	**children**	**racist**	**disabled**	**gop**	fake	**public**	**internet**
	**voted**	**poor**	florida	customer	affiliate	idiot	worker
	fox	**republican**	ibm				
**Clustr3**	**tip**	**aphasia**	**survivor**	**stroke survivor**	**amazon**	**tweet**	**rehab**
	presse	kindle	newsletter	conquer	monthly	Chance win	
**Clust4**	**pop**	**movie**	**blog**	**video**	**eye**	**tube**	english
	**playlist**	exclusive	stroke blog				
**Clust5**	**hear**	symptoms	**women**	**cancer**	**drug**	**heart**	mile walk
	**surgery**	**death**	stroke aphasia				
**Cl6**	**follower**	stat	**twitter**	**retweet**	arrived	twitting	unfollower
	unfollowed	today stat					
**Cl7**	brain injury	earning	**info**	killer	**awareness**	**war**	iphone
	**phone**	regional					

Table A2 Tabular representation of Women clusters obtained with VosViewer

**Cluster1**	hug	wow	**awesome**	mum	**fan**	**player**	**mom**
	shopping	**tomorrow**	**lol**	**amazing**	omg	**girl**	**friend**
	**kick**	**love**	**tough**	**kid**	miss	fab	**bike**
	**sun**	**film**	**episode**	chocolate	**ticket**	**football**	hun
	wanna	fabulous	weird	**fantastic**	boutique	**phone**	hello
	definitely	hopefully	**hard**	**head**	concert	shoe	chicken
	haha	hubby	**rip**	yeah	recipe	**dad**	**morning**
	**happy birthday**	**pic**	sweetie	anymore	**gotta**	**gonna**	cute
	**pop**	gorgeous	**toge**	**night**	**tonight**	**door**	sat
	**movie**	**lady**	bably	**super**	**weekend**	aww	velvette
**Cluster 2**	**public**	conver	**veteran**	**water**	**voice**	scorpio	**business**
	**power**	investig	**radio**	kavanaugh	**voted**	**biggest**	voting
	**congress**	**air**	**adult**	**house**	**hear**	**surgery**	**fear**
	educ	**country**	**poor**	**cancer**	**lon**	**wife**	emotional
	**disabled**	**white**	photoset	**lord**	**speak**	**bus**	psalm
	**fire**	mike	son	**god**	**drug**	**city**	**racist**
	**women**	symptom	**died**	**gop**	**heart**	mise	**republican**
	**money**	cont	bro	**law**	**disability**	**brexit**	already discovered
	**woman**	**bbc**	**child**	**trump**	intern	answer	
	**children**	**democrat**	senate	**eye**	**obama**	**death**	
**Cluster 3**	**stroke survivor**	**tour**	**announce**	**war**	**volunteer**	**tweet**	hey
	inspiring	**massive**	**aphasia**	**congratul**	**survivor**	**awareness**	inspir
	**follower**	**journey**	chat	**award**	**read**	**winning**	**congrat**
	**giveaway**	**rehab**	professional	**info**	**amazon**	**retweet**	**twitter**
	marathon	**website**	**tip**	**blog**	**winner**	exciting	mommy
	dvd						
**Clust4**	wish	**thursday**	ppl	**birthday**	halloween	wednesday	celebrating
	**body**	**tuesday**	stafford	**anniversary**	**celebrate**	**app**	
**Cl5**	**internet**	**launch**	**photo**	**facebook**	**video**	**pal**	**album**
	vide	**playlist**	**google**	**tube**			

Words in **bold** are those that belong to both men and women clusters, therefore we analyzed only not bold words with hedonometer

Selection of words only present in men clusters (top) and women cluster(bottom) with their respective hedonometer scores:

Table A3 words only present in men clusters and their hedonometer scores

**Clur 1**	league (5.80)	dude (5.80)	manager (5.02)	baseball (5.84)	daily (5.4) photo (6.88)	gym (5.96)	Manchester (5.56)
	damn (2.98)	couldn't (3.58)	stadium (5.96)	nfl (5.16)	happening (5.72)	entry (5.74)	sponsor (6.00)
	England (6.28)	Arsenal (4.18)	land (6.18)	instagram	buddy (6.84)		
**Clusr 2**	petition (4.80)	poll (5.62)	golf (5.56)	fake (2.90)	USA (6.58)	wouldn (4.40)	worker (5.56)
	funding	conservative (4.54)	doubt (3.16)	canadian (5.84)	supporter (6.76)	shouldn (3.84)	idiot (3.06)
	corbyn	illegal (2.86)	Florida (6.70)	customer (5.66)	affiliate (5.68)	ibm (5.58)	tech (6.22)
	fox (5.46)	clinton (5.68)					
**C3**	presse (5.36)	kindle (6.14)	newsletter	conquer	monthly (5.42)	chance (6.44) win (8.12)	
**C4**	exclusive (6.48)	stroke (2.58) blog (6.02)	English (6.58)				
**C5**	stroke (2.58) aphasia	symptoms (3.76)	mile (5.04) walk (6.02)				
**C6**	unfollowed	stats (5.28)	arrived (6.14)	Twitting (5.46)	Today (6.22) stats (5.28)	unfollower	
**C7**	brain (6.52) injury (2.04)	earning (6.94)	regional (5.34)	killer (2.42)	iphone (6.54)		

Table A4 words only present in women clusters and their hedonometer scores

**Clusr1**	hug (7.58)	wow (7.46)	concert (6.94)	mum (6.70)	boutique	recipe (6.50)	cute (7.52)
	shopping (7.10)	bably	aww (5.12)	velvette (6.51)	omg (5.10)	shoe (5.84)	sat (5.24)
	wanna (5.50)	hubby (6.16)	gorgeous (7.42)	yeah (5.90)	miss (3.64)	fab (6.10)	hun (5.08)
	definitely (6.20)	sweetie (7.30)	weird (4.20)	chocolate (7.86)	chicken (6.40)	hello (7.00)	haha (7.64)
	fabulous (7.38)	hopefully (6.84)	anymore (4.56)				
**Clur 2**	educ (6.88)	conver	photoset	bro (6.22)	mise	scorpio	cont
	mike	Investigate (5.10)	son (7.12)	kavanaugh	already (5.28) discovered (7.00)	emotional (4.42)	voting (6.02)
	symptom (3.76)	senate (4.70)	intern	answer (6.24)	psalm		
**Cl 3**	exciting (7.58)	mommy (7.42)	Halloween (6.24)	Wednesday (5.38)	celebrating (8.00)	inspire (7.12)	hey (6.06)
	Inspiring (7.34)	chat (6.42)	Professional (6.44)	dvd (6.66)	marathon (5.56)		
**C4**	Wish (6.92)	Stanford (5.90)	ppl (5.54)				

Table A5. Mean hedonometer scores

	**Men**	**Women**
	Mean ± sd	Mean ± sd
total	5.31±1.31	6.25 ±1.07
Cluster 1	5.52 ±0.99	6.30 ± 1.12
Cluster 2	5.06±1.25	5.70± 1.13

LDA topic models

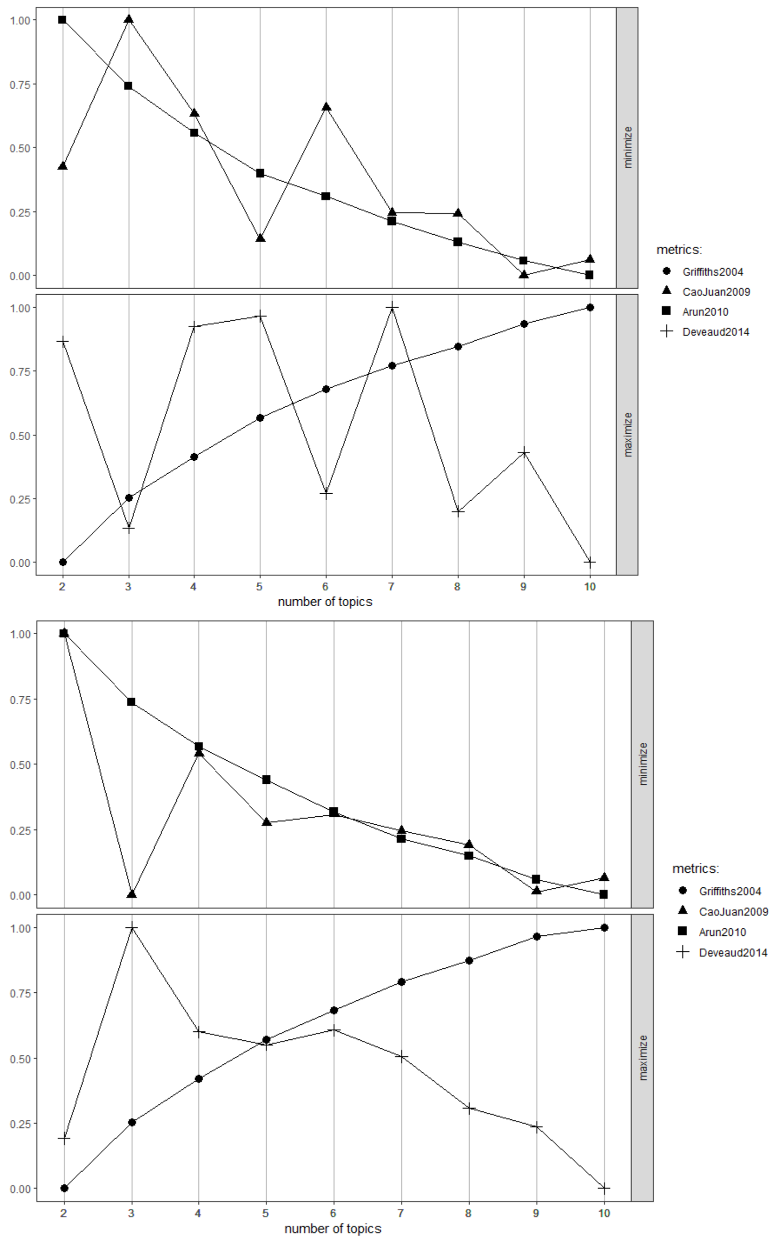

Figure A4. Criteria for optimal number of topics k=7 is optimal (women up, men bottom)

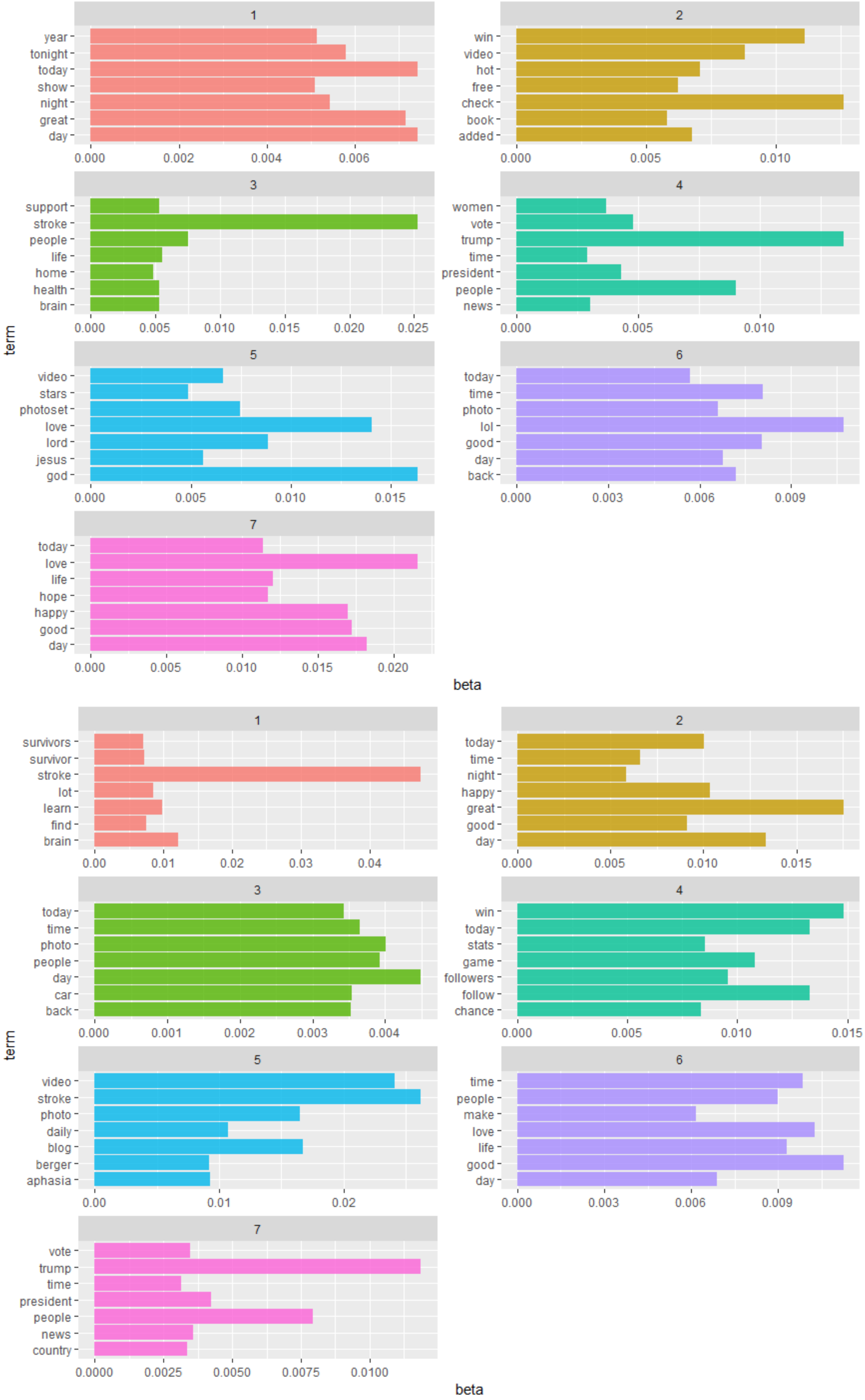

Fig A5 LDA obtained topic models women up, men bottom

Correlation analysis within topics

We conducted correlation analysis with pairs of words, for each topic we took pairs of the 6 words and plotted all the words most highly correlated to each of them, in order to provide us more elements to be able to interpret the topic. As shown in the plot below, words most highly correlated to "photo" are "daily", "posted", "instagram" and "facebook"; words correlated to "car" are "nearby" or "skidded".

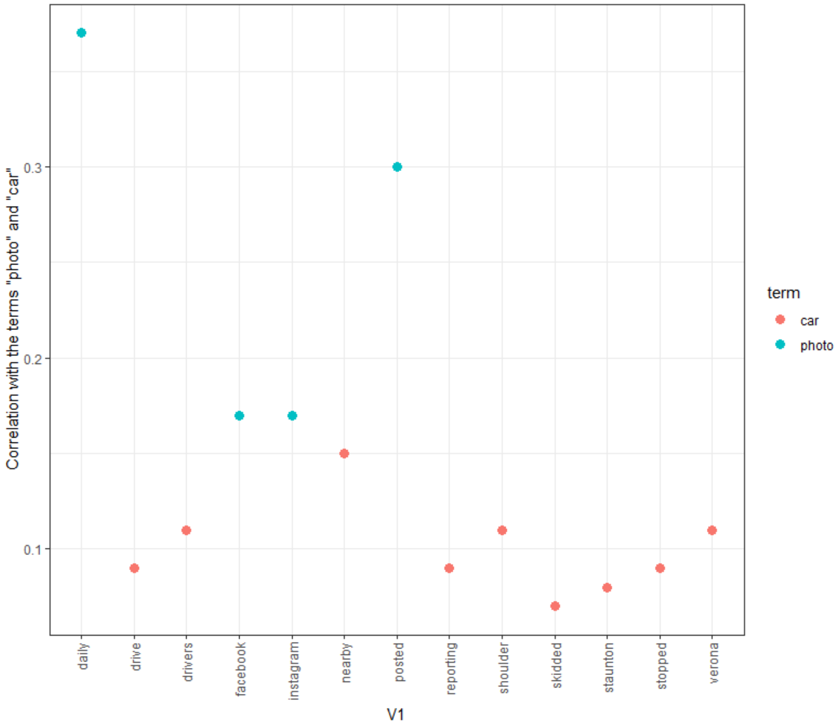

STM

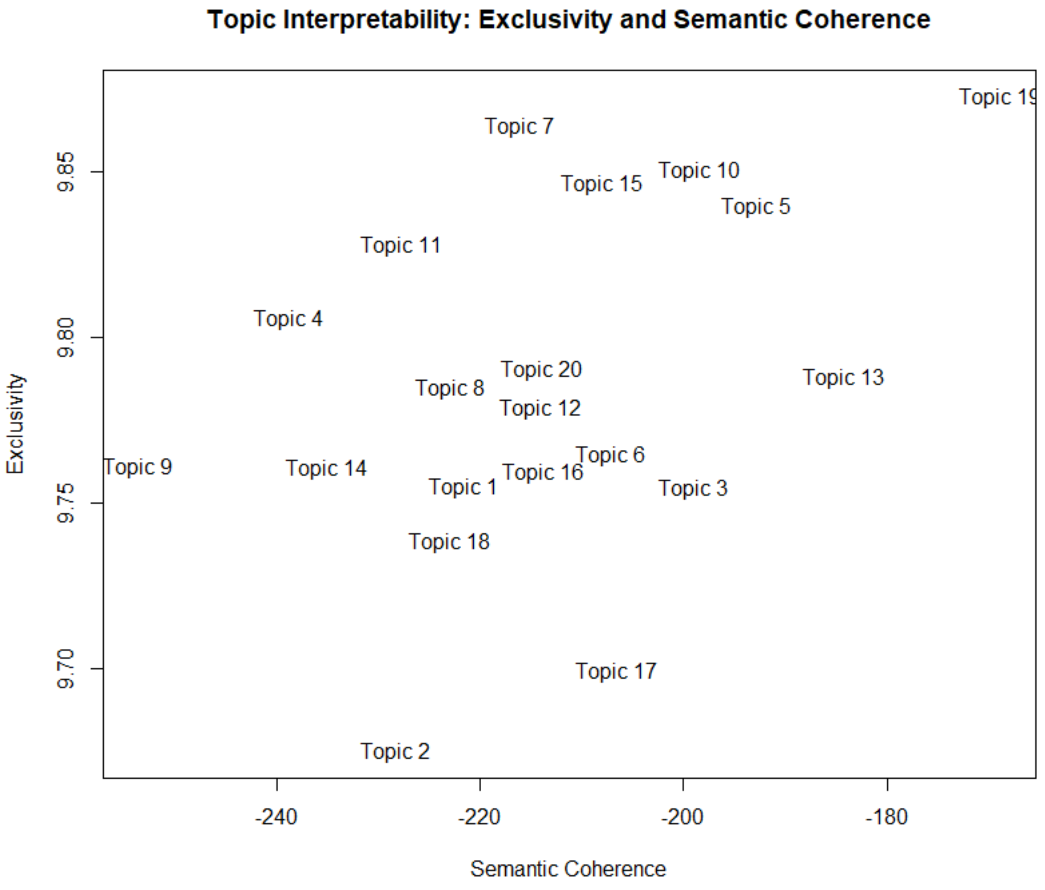

Fig A6 Evaluation of STM obtained topics

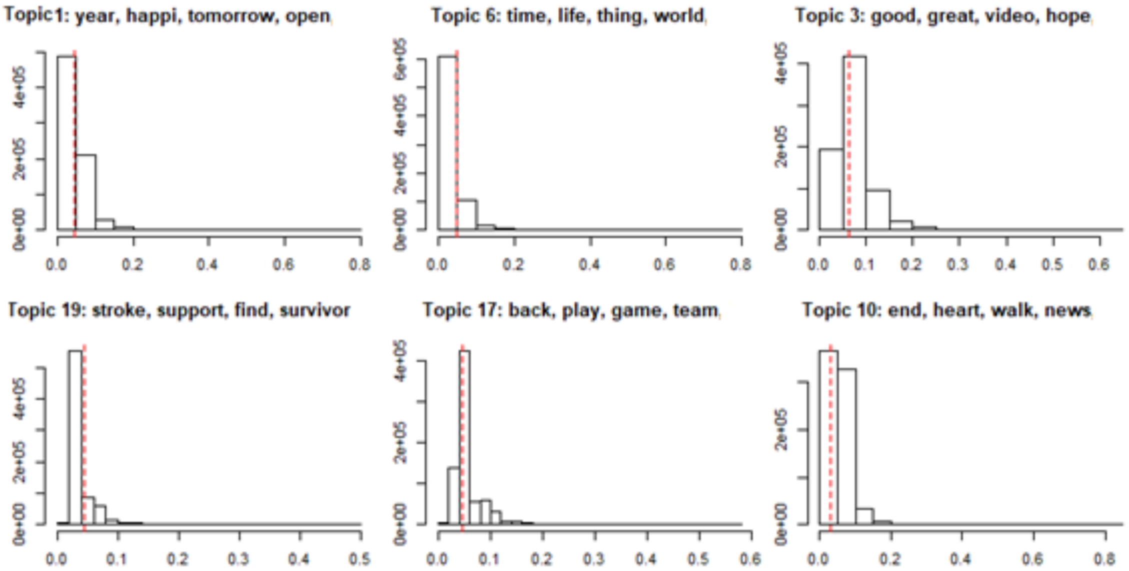

Fig 7A Frequencies of the selected 6 topics

Table A6. STM 20 Topics and their corresponding percentage

**Topic 1 (5.23%)**	
Highest Prob	year, happy, tomorrow, open, birthday, take, come, busy, christmas, baby, sleep, friday, sunday, monday, list, bed, smile, market, treat, guess
FREX	merri, birthday, appl, ang, eve, awak, con, clay, decemb, angel, happi, est, syracus, ako, relax, andrea, closet, lang, store, carousel
Score	happi, year, birthday, tomorrow, open, christma, sleep, friday, come, busi, babi, sunday, take, bed, store, monday, holiday, list, date, market
**Topic 2 (4.31%)**	
Highest Prob	listen, music, song, review, kind, young, write, reason, view, son, challeng, order, dad, case, daughter, art, updat, email, brilliant, shot
FREX	bowl, semua, sama, bersama, disini, temukan, keseruan, santai, ada, amanz, luton, nicola, itu, aku, yang, poem, cultur, tapi, livelyr, aja
Score	song, listen, music, review, write, son, view, challeng, art, dad, kind, reason, brilliant, email, semua, sama, order, bersama, keseruan, temukan
**Topic 3 (7.21%)**	
Highest Prob	good, great, video, hope, night, morn, lol, tonight, long, done, head, weekend, fun, readi, celebrate, citi, movi, luck, earli, forget
FREX	playlist, chicken, grill, peter, egg, chees, movi, delici, potato, cooki, bbq, soup, recip, video, kitti, cup, chili, luck, pan, belli
Score	good, video, hope, night, morn, great, lol, weekend, playlist, movi, tonight, don, luck, fun, long, sweet, forget, gonna, saturday, dinner
**Topic 4 (4.16%)**	
Highest Prob	check, blog, school, save, high, servic, interest, hey, hot, problem, product, complet, memori, improv, pain, perform, action, compani, base, system
FREX	product, richard, walker, boutiqu, hot, loan, mood, kendal, jackson, hey, recommend, modern, massiv, syndrom, envi, portland, usda, payment, harri, outstand
Score	check, blog, product, hot, school, hey, servic, boutiqu, save, richard, high, problem, improv, walker, pain, interest, compani, action, perform, activ
**Topic 5 (5.87%)**	
Highest Prob	day, today, follow, week, friend, miss, person, beauti, guy, awesom, found, set, unfollow, daili, wonder, stat, number, past, glad, rest
FREX	unfollow, stat, past, taurus, stafford, automat, lake, spring, san, farm, tweep, trick, ray, goodby, ibm, francisco, virginia, crunch, peep, pub
Score	day, today, follow, unfollow, week, stat, friend, miss, awesom, person, beauti, daili, guy, found, past, wonder, grow, glad, number, goal
**Topic 6 (5.73%)**	
Highest Prob	time, life, thing, world, god, famili, twitter, hear, power, hate, pass, speak, human, step, posit, bless, super, continu, messag, creat
FREX	god, lord, pray, faith, amen, bless, prayer, psalm, soul, negat, holi, heal, thank, charl, nchousingbuild, merci, evil, accomplish, yea, compass
Score	time, god, thing, life, famili, twitter, world, lord, bless, hear, power, step, super, hate, pray, prayer, congrat, pop, faith, posit
**Topic 7 (4.77%)**	
Highest Prob	photo, post, read, stori, talk, share, join, train, excit, latest, face, facebook, inspir, event, idea, survivor, men, half, radiant, gazett

FREX	latest, radiant, gazett, facebook, dysplasia, fibromuscular, bus, blood, baro, yonker, cox, writer, express, workshop, post, photo, mta, marathon, ted, arteri
Score	photo, post, read, radiant, gazett, latest, stori, facebook, talk, share, join, survivor, train, excit, event, blood, inspir, idea, face, funni
**Topic 8 (3.91%)**	
Highest Prob	big, report, hit, stand, question, deal, public, attack, told, media, fire, side, fall, social, lead, cover, ask, tell, sale, investig
FREX	journal, wheel, fortun, media, jeremi, aaron, robert, prime, oil, jone, entri, minist, carolina, bond, roger, secret, general, jay, chick, hit
Score	big, report, hit, media, deal, stand, attack, social, journal, public, entri, told, question, investig, side, fire, brexit, fall, lead, tell
**Topic 9 (4.00%)**	
Highest Prob	put, didn, fan, star, dog, link, wow, line, cut, unit, turn, poor, hell, rain, forc, ball, univers, ice, aren, shirt
FREX	popular, feed, sold, bye, christin, sock, yellow, mirror, credit, cream, sharon, ice, heavi, apart, horror, chase, cabl, figur, muqoda, piti
Score	didn, dog, star, link, wow, fan, put, cut, line, rain, ice, hell, unit, ball, poor, forc, univers, shirt, add, figur
**Topic 10 (3.82%)**	
Highest Prob	end, heart, walk, news, stop, run, hand, pay, mile, rate, worth, success, dead, offer, singl, reach, staff, fail, snow, hero
FREX	mile, rate, bpm, attitud, stephani, anthem, bioness, hawk, failur, shoulder, flaw, casual, complic, tattoo, zombi, hero, pinterest, hand, virus, vancouv
Score	heart, walk, end, news, mile, stop, run, rate, bpm, hand, pay, dead, success, attitud, hero, snow, worth, bbc, offer, reach
**Topic 11 (3.84%)**	
Highest Prob	book, free, chang, paul, age, aphasia, phone, issu, manag, result, articl, receiv, aign, strong, app, berger, sun, mental, fit, due
FREX	aphasia, result, articl, sever, paralysi, spell, guid, shine, kati, lab, hip, mommi, nasa, upgrad, woo, hop, hoo, ace, app, tornado
Score	book, free, paul, aphasia, berger, chang, phone, result, age, articl, issu, manag, app, websit, guid, prevent, sun, strong, jesus, mental
**Topic 12 (5.41%)**	
Highest Prob	work, home, made, rememb, hard, hour, left, stay, leav, agre, kill, sad, food, car, buy, eat, offic, drive, total, water
FREX	burn, min, calori, eat, jerri, workout, meal, toilet, road, hour, plant, thankyou, cheap, ben, safe, frozen, snack, freez, carson, whale
Score	work, home, made, hour, hard, food, stay, eat, left, leav, kill, agre, rememb, sad, total, road, drive, offic, water, burn
**Topic 13 (5.72%)**	
Highest Prob	trump, call, vote, part, women, presid, countri, break, state, american, parti, white, lie, million, polic, america, govern, elect, fact, member
FREX	presid, obama, donald, republican, elect, democrat, pal, gop, sexual, senat, racist, assault, law, crime, immigr, trump, rape, voter, poll, accus
Score	trump, vote, presid, state, american, call, countri, elect, part, lie, women, white, obama, america, govern, parti, break, law, donald, republican
**Topic 14 (4.96%)**	

Highest Prob	make, show, word, true, hous, top, bring, visit, pictur, shop, wear, pic, build, rock, race, page, discov, onlin, releas, begin
FREX	discov, locat, rts, episod, podcast, hashtag, lmao, visitor, church, ador, nope, site, shazam, graduat, prom, fave, baton, louisiana, ident, wilson
Score	make, show, word, true, discov, top, locat, bring, pic, pictur, hous, shop, wear, site, episod, rock, visit, page, click, spread

**Topic 15 (4.38%)**	
Highest Prob	win, sign, final, enjoy, chanc, retweet, season, forward, close, enter, proud, finish, ticket, eye, gift, special, pick, award, want, winner
FREX	win, enter, chanc, winner, giveaway, prize, pack, card, copi, ticket, contest, competit, sticker, gee, code, jersey, coupon, awww, draw, tenni
Score	win, chanc, enter, retweet, sign, enjoy, season, final, ticket, gift, winner, giveaway, forward, card, finish, award, proud, pick, pack, special
**Topic 16 (5.54%)**	
Highest Prob	love, watch, live, tweet, amaz, bad, man, nice, full, mind, fight, sound, yesterday, lon, moment, pretti, red, interview, leg, street
FREX	leg, lbs, press, cute, street, voga, photographi, rio, cheer, acquaint, android, coin, pool, las, nice, carpet, nicol, mad, smith, columbia
Score	love, watch, live, tweet, nice, amaz, full, leg, bad, lon, press, street, fight, pretti, yesterday, sound, lbs, mind, moment, red
**Topic 17 (5.25%)**	
Highest Prob	back, play, game, team, job, place, boy, man, point, won, lost, black, park, perfect, act, lose, john, footbal, film, player
FREX	yard, hole, playoff, player, joe, nfl, eagl, cowboy, bronx, kiss, dalla, theater, doodl, lewi, cunt, throw, golden, barn, korea, brave
Score	game, team, play, back, boy, job, footbal, player, park, perfect, black, act, place, north, beat, test, film, lose, tour, kick
**Topic 18 (5.18%)**	
Highest Prob	peopl, feel, give, happen, move, die, plan, nation, fuck, disabl, wrong, minut, understand, woman, matter, offici, mean, heard, death, shit
FREX	der, und, muse, die, ein, bookworm, hurrican, ist, opinion, von, alright, bin, das, auf, nasti, rico, shut, puerto, ich, twist
Score	peopl, feel, die, give, happen, fuck, move, disabl, plan, wrong, minut, understand, matter, nation, woman, truth, deserv, death, photoset, offici
**Topic 19 (6.25%)**	
Highest Prob	stroke, support, find, survivor, learn, lot, brain, care, health, patient, help, awar, money, rais, children, research, import, risk, experi, hospit
FREX	awar, rais, foundat, risk, research, patient, region, medic, lot, recoveri, donat, increas, factor, studi, resourc, rehab, treatment, cancer, rehabilit, learn
Score	stroke, survivor, learn, lot, find, support, brain, patient, awar, care, rais, health, research, risk, foundat, injuri, studi, region, recoveri, diseas
**Topic 20 (4.44%)**	
Highest Prob	start, wait, thought, meet, kid, month, girl, real, ago, bit, send, think, earn, haha, mom, cool, absolut, dream, tip, stuff
FREX	reward, sarah, subscrib, newslett, blue, send, psychic, halloween, builder, math, santa, form, omg, yup, vocab, girl, ferri, channel, nap, haha

Score	girl, month, meet, thought, earn, wait, kid, send, real, bit, tip, haha, blue, think, mom, start, cool, ago, dream, absolut

Hedonomter : happiness scores assigned to all words in topics

Table A7. Complete list of words for each topic and happiness score by gender (women violet, men light blue)

Word	Score	Topic	Word	Score	Topic	Word	Score	Topic	Word	Score	Topic
monday	4.3	T1	hate	2.34	T6	aphasia	2.58	T11	bad	2.64	T16
busy	4.58	T1	step	5.32	T6	due	4.22	T11	fight	2.7	T16
take	5.18	T1	twitter	5.46	T6	issue	4.48	T11	lon	4.74	T16
year	5.38	T1	thing	5.58	T6	mental	4.8	T11	yesterday	4.98	T16
list	5.46	T1	time	5.74	T6	age	4.88	T11	interview	5.06	T16
guess	5.64	T1	continue	5.76	T6	berger	5.08	T11	street	5.44	T16
come	5.82	T1	hear	5.84	T6	app	5.2	T11	red	5.56	T16
open	6.1	T1	pass	5.9	T6	paul	5.44	T11	leg	5.62	T16
tomorrow	6.18	T1	speak	5.9	T6	article	5.52	T11	moment	5.68	T16
market	6.28	T1	message	6.08	T6	change	5.56	T11	watch	5.7	T16
treat	6.74	T1	human	6.26	T6	sign	5.66	T11	tweet	5.84	T16
friday	6.88	T1	world	6.52	T6	manage	5.74	T11	sound	5.86	T16
sleep	7.16	T1	power	6.68	T6	result	6	T11	man	5.9	T16
bed	7.18	T1	create	6.82	T6	fit	6.22	T11	mind	6.04	T16
baby	7.28	T1	bless	7.08	T6	phone	6.44	T11	full	6.29	T16
sunday	7.28	T1	god	7.28	T6	receive	6.5	T11	live	6.9	T16
birthday	7.78	T1	life	7.32	T6	strong	7.06	T11	pretty	7.32	T16
christmas	7.96	T1	super	7.68	T6	book	7.24	T11	nice	7.38	T16
smile	8.1	T1	family	7.72	T6	sun	7.8	T11	amazing	7.66	T16
happy	8.3	T1	positive	7.8	T6	free	7.96	T11	love	8.42	T16
shot	2.5	T2	half	4.6	T7	kill	1.56	T12	lost	2.76	T17
case	5.02	T2	post	5.48	T7	sad	2.38	T12	lose	2.76	T17
order	5.22	T2	men	5.94	T7	hard	4.1	T12	black	4.88	T17
review	5.32	T2	train	5.98	T7	leave	4.52	T12	john	5.08	T17
reason	5.54	T2	talk	6.06	T7	left	4.64	T12	back	5.18	T17
view	5.72	T2	latest	6.06	T7	office	5.12	T12	point	5.42	T17
update	5.72	T2	facebook	6.08	T7	work	5.24	T12	place	5.56	T17
challenge	5.78	T2	face	6.12	T7	hour	5.32	T12	man	5.9	T17
write	5.8	T2	event	6.12	T7	stay	5.56	T12	player	5.94	T17
listen	5.94	T2	join	6.22	T7	total	5.78	T12	job	5.96	T17
art	6.6	T2	gazett	6.32	T7	made	5.96	T12	act	6	T17
young	6.82	T2	story	6.48	T7	drive	6.08	T12	boy	6.24	T17
email	6.88	T2	read	6.52	T7	buy	6.28	T12	team	6.26	T17

dad	7.02	T2	survivor	6.66	T7	remember	6.42	T12	footbal	6.34	T17
daughter	7.04	T2	share	6.78	T7	agree	6.7	T12	film	6.56	T17
son	7.12	T2	photo	6.88	T7	water	6.7	T12	game	6.92	T17
kind	7.24	T2	radiant	7.04	T7	car	6.72	T12	park	7.08	T17
song	7.58	T2	idea	7.06	T7	eat	7.04	T12	play	7.26	T17
brilliant	7.68	T2	inspire	7.12	T7	home	7.14	T12	perfect	7.42	T17
music	8.02	T2	exciting	7.58	T7	food	7.44	T12	won	8.1	T17
forget	3.22	T3	attack	2.42	T8	lie	2.6	T13	death	1.54	T18
morn	4.36	T3	fire	3.8	T8	police	4.58	T13	die	1.74	T18
long	4.74	T3	fall	4.04	T8	part	4.98	T13	shit	2.5	T18
early	5.66	T3	hit	4.58	T8	parti	5	T13	disabled	2.82	T18
city	5.76	T3	report	4.76	T8	break	5.04	T13	wrong	3.14	T18
head	5.96	T3	question	4.98	T8	govern	5.1	T13	mean	3.68	T18
tonight	6.14	T3	told	5.08	T8	state	5.28	T13	fuck	4.14	T18
night	6.22	T3	investigate	5.1	T8	trump	5.3	T13	happen	5.28	T18
video	6.48	T3	side	5.12	T8	call	5.74	T13	heard	5.36	T18
done	6.54	T3	public	5.18	T8	vote	5.8	T13	official	5.38	T18
ready	6.58	T3	ask	5.2	T8	president	5.86	T13	matter	5.4	T18
lol	6.84	T3	cover	5.32	T8	fact	5.9	T13	minute	5.5	T18
movie	6.84	T3	tell	5.42	T8	member	5.94	T13	move	5.72	T18
good	7.2	T3	stand	5.6	T8	elect	6.1	T13	feel	5.9	T18
luck	7.26	T3	media	5.62	T8	country	6.28	T13	nation	6.06	T18
hope	7.38	T3	big	6.22	T8	white	6.28	T13	plan	6.14	T18
great	7.88	T3	social	6.22	T8	american	6.74	T13	people	6.16	T18
fun	7.96	T3	lead	6.28	T8	america	6.76	T13	give	6.54	T18
celebrate	7.98	T3	deal	6.32	T8	women	7.12	T13	understand	6.56	T18
weekend	8	T3	sale	6.58	T8	million	7.38	T13	woman	6.84	T18
pain	2.1	T4	hell	2.22	T9	bring	5.3	T14	stroke	2.58	T19
problem	2.98	T4	poor	2.32	T9	word	5.56	T14	hospital	3.5	T19
hot	5.02	T4	cut	3.42	T9	page	5.62	T14	risk	3.62	T19
base	5.08	T4	force	4	T9	race	5.76	T14	patient	5.04	T19
system	5.3	T4	arent	4.34	T9	wear	5.94	T14	import	5.72	T19
company	5.44	T4	didn	4.8	T9	pic	5.98	T14	experiment	5.76	T19
product	5.56	T4	line	4.98	T9	make	6	T14	lot	5.78	T19
action	5.98	T4	put	5.04	T9	release	6.1	T14	aware	5.78	T19
service	6	T4	turn	5.2	T9	rock	6.14	T14	find	6	T19
blog	6.02	T4	unit	5.5	T9	build	6.22	T14	help	6.08	T19
hey	6.06	T4	link	5.78	T9	show	6.24	T14	raise	6.36	T19
check	6.1	T4	ice	5.8	T9	begin	6.28	T14	support	6.44	T19
school	6.26	T4	ball	6.08	T9	house	6.34	T14	research	6.46	T19
interest	6.36	T4	shirt	6.26	T9	visit	6.54	T14	brain	6.52	T19
improve	6.44	T4	rain	6.36	T9	discovery	6.54	T14	care	6.64	T19
memory	6.46	T4	fan	6.66	T9	picture	6.64	T14	survivor	6.66	T19

perform	6.48	T4	dog	6.7	T9	online	6.72	T14	learn	6.7	T19
save	6.54	T4	universe	6.84	T9	top	6.76	T14	children	7.12	T19
high	6.64	T4	star	7.22	T9	shop	6.76	T14	health	7.22	T19
complete	6.74	T4	wow	7.46	T9	true	7.08	T14	money	7.3	T19
miss	3.64	T5	fail	1.96	T10	close	4.94	T15	wait	3.74	T20
unfollow	4.06	T5	dead	2	T10	final	5.02	T15	bit	4.54	T20
past	4.62	T5	stop	3.9	T10	retweet	5.12	T15	ago	4.88	T20
week	5.08	T5	end	4.36	T10	ticket	5.32	T15	month	5.18	T20
stats	5.28	T5	mile	5.04	T10	pick	5.6	T15	send	5.38	T20
number	5.3	T5	single	5.12	T10	sign	5.66	T15	absolute	5.4	T20
daily	5.4	T5	pay	5.3	T10	want	5.7	T15	stuff	5.58	T20
person	5.54	T5	rate	5.34	T10	enter	5.84	T15	start	6.1	T20
set	5.58	T5	staff	5.36	T10	season	5.96	T15	think	6.2	T20
follow	5.66	T5	run	5.48	T10	forward	6.1	T15	tip	6.24	T20
guy	5.92	T5	news	5.6	T10	eye	6.14	T15	thought	6.38	T20
today	6.22	T5	reach	5.74	T10	chance	6.44	T15	meet	6.38	T20
day	6.24	T5	hand	5.9	T10	finish	6.72	T15	real	6.78	T20
found	6.54	T5	walk	6.02	T10	special	7.2	T15	kid	6.84	T20
wonder	7.08	T5	snow	6.32	T10	proud	7.32	T15	girl	7	T20
rest	7.18	T5	offer	6.62	T10	enjoy	7.66	T15	dream	7.1	T20
glad	7.48	T5	worth	6.78	T10	gift	7.72	T15	cool	7.2	T20
awesome	7.6	T5	heart	7.22	T10	winner	7.78	T15	earn	7.5	T20
friend	7.66	T5	hero	7.8	T10	award	7.86	T15	haha	7.64	T20
beauty	7.76	T5	success	7.86	T10	win	8.12	T15	mom	7.64	T20

Statistical comparisons

We then statistically compared (t test) the means of the scores obtained in each pair of topics, considering pairs placed in the same position of Figure 6 (i.e. the pair Topic 19 and Topic 1 because they are both at the extremes of Figure 6, then Topic 17 with Topic 6, etc). All comparisons are shown in the figure below.

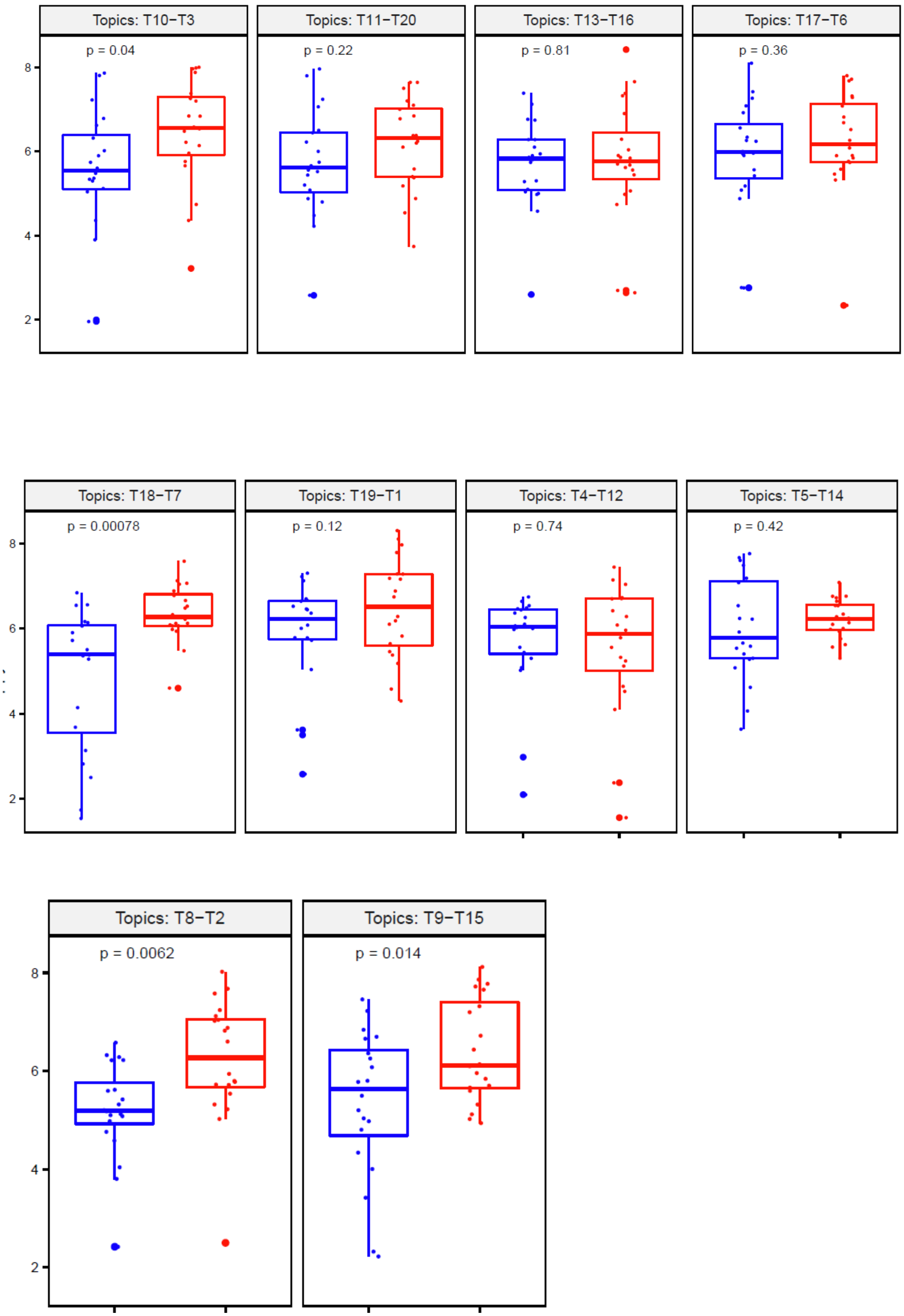

Figure 8A. Comparisons of happiness scores for corresponding topics

Plutchik model

Positive and negative valences are associated to emotions based on Plutchik's Psychoevolutionary Model of the Primary Emotions. (TenHouten W.D. 2017).
Plutchik, developed a psychoevolutionary model in which he identified four life problems: identity, temporality, hierarchy, and territoriality.
Each of these life problems can be either an opportunity or a danger, so that a situation is either negatively or positively valenced.
Any of the eight problem-valence situations can therefore occur, each of which triggers a distinct subjective state of mind which activates an adaptive reaction. These eight prototypical adaptive reactions, constitute the 8 primary emotions, associated with positive and negative valences as shown in the table below:

**Life problems**	**Basic functions**	**Emotions**	**Valences**
Identity	Incorporation	Trust (Acceptance)	Positive
	Rejection	Disgust	Negative
Temporality	Reproduction	Joy	Positive
	Reintegration	Sadness	Negative
Hierarchy	Destruction	Anger	Positive
	Protection	Fear	Negative
Territoriality	Exploration	Anticipation	Positive
	Boundary defense	Surprise	Negative

It might seem counterintuitive to consider anger a positive emotion, and indeed several researchers have assigned it a negative valence because it is associated with unpleasant eliciting situations (Ekman and Davidson 1994; Ekman and Friesen 1975) and occurs in situations that are incongruent with one's goals (Lazarus 1991). But anger *is* positive in that it is an emotion that evokes behavioral tendencies of approach and is associated with attack behavior, functioning to move aside, or destroy or move aside, an obstacle standing in the way of a goal, which is an assertive course of action (Darwin 1872; Plutchik 1980). Fear is a self-protective response of moving away from a dangerous or problematic person or situation, which is clearly of negative valence insofar as it is both unpleasant and avoidant motivated.

References

TenHouten W.D. (2017) From Primary Emotions to the Spectrum of Affect: An Evolutionary Neurosociology of the Emotions. In: Ibáñez A., Sedeño L., García A. (eds) Neuroscience and Social Science. Springer
